# Novel loop-like aromatic compounds: a further step on the road to nanobelts and nanotubes

**DOI:** 10.3762/bjoc.6.30

**Published:** 2010-03-29

**Authors:** Venkataramana Rajuri, Dariush Ajami, Gaston R Schaller, Christian Näther, Rainer Herges

**Affiliations:** 1 Otto-Diels-Institut für Organische Chemie, Christian-Albrechts-Universität Kiel, Otto-Hahn-Platz 4, 24098 Kiel, Germany; 2 Otto-Diels-Institut für Organische Chemie, current address: The Skaggs Institute for Chemical Biology and Department of Chemistry, The Scripps Research Institute, 10550 North Torrey Pines Road, La Jolla, CA 92037, U.S.; 3 Institut für Anorganische Chemie, Christian-Albrechts-Universität Kiel, Otto-Hahn-Platz 6/7, 24098 Kiel, Germany

**Keywords:** [*n*]annulenes, Diels–Alder reaction, nanobelts, tetradehydrodianthracene

## Abstract

The synthesis and crystal structural characterization of new compounds **2–6** were accomplished. As a common synthetic methodology, the Diels–Alder reaction was applied to 9,9′,10,10′-tetradehydrodianthracene (TDDA) (**7**) to furnish the [12]annulenes **2** and **3** [16]annulene **6** and adduct **5**.

## Introduction

The first loop-like (in plane) conjugated molecules [1–2] prepared by rational synthesis were reported 13 years ago [3–5]. Unlike “normal” cyclic conjugated rings with p orbitals perpendicular to the ring plane such as benzene, the p orbitals of belt-like conjugated structures are orthogonal with respect to the surface of a cylinder and the inner phases of the p orbitals point to the axis of the system. Whereas cyclic anthracenylidenes and nanorings consist of a single stranded path of cyclic conjugated C-C bonds, the first double stranded conjugated belt was described only recently by Gleiter et al. [6]. These molecular belts are composed of an alternating sequence of four- and eight-membered rings. Belts that exclusively consist of benzene rings, such as cyclacenes [7–8] are still elusive. These molecules are very interesting targets because they are substructures of carbon nanotubes which could eventually be extended in length by chemical vapor deposition [9–10]. This strategy could eventually open a way to prepare monodisperse nanotubes with well defined physical properties.

Our approach to synthesize loop-like aromatic compounds is based on the Diels–Alder reaction [7–8,11] and ring enlargement metathesis [12–15] of 9,9′,10,10′-tetradehydrodianthracene (TDDA) (**7**) [16]. In a second step we anticipated closing the tube walls by cyclodehydrogenation [17].

## Results and Discussion

We now report on the synthesis of five new compounds, two hydrocarbons, two halogenated derivatives and one silver complex, which with reference to the definitions of Scott [1] and Tobe [18], belong to the class of bridged *all*-*Z*-[*n*]annulenes, which are a subclass of loop-like or single-stranded molecules [19]. All structures were characterized by crystal structure analysis.

**Scheme 1:**
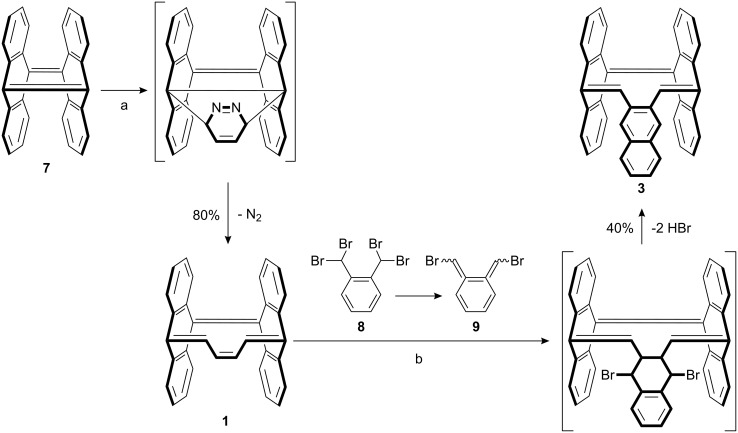
Synthesis of **1** and **3**. (a) Pyridazine, toluene, reflux, 20 h; (b) NaI, DMF, 65 °C, 15 h.

**Scheme 2:**
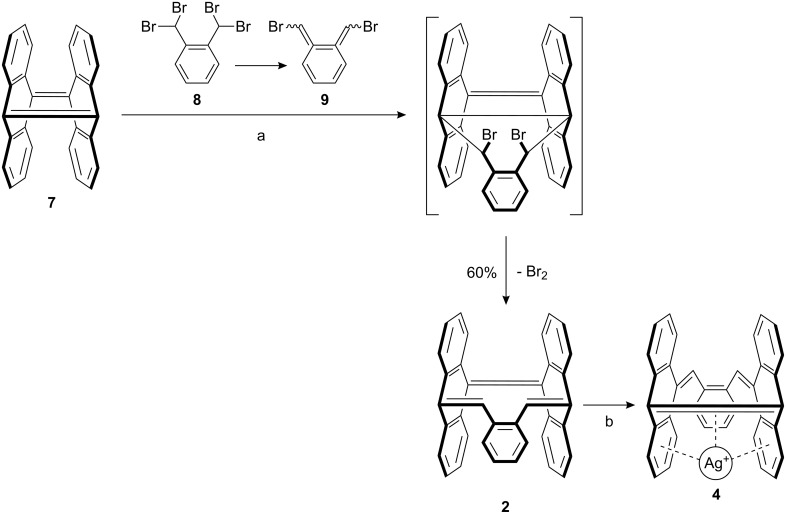
Synthesis of **2** and **4**. (a) NaI, DMF, 65 °C, 24 h; (b) AgSbF_6_, THF.

The pure hydrocarbons **2** and **3** were prepared by Diels–Alder reaction of either **7** or **1** [20] with 1,2-bis(dibromomethyl)benzene (**8**) and sodium iodide to furnish the desired compounds, **2** and **3**, in 60 and 40% yield, respectively (Scheme 1 and Scheme 2).

During this simple one pot reaction, the sodium iodide induces a 1,4-elimination reaction of **8** resulting in the formation of the highly reactive quinodimethane 5,6-bis(bromomethylene)cyclohexa-1,3-diene (**9**). Diene **9** and the central olefinic double bond of **1** react in a Diels–Alder reaction, followed by a double dehydrobromination, to afford the desired product **3**. The reaction of diene **9** with the quinoid bridgehead double bond of **7** gives **2**. In this case the Diels–Alder reaction is followed by a debromination in combination with an electrocyclic ring opening.

**Figure 1:**
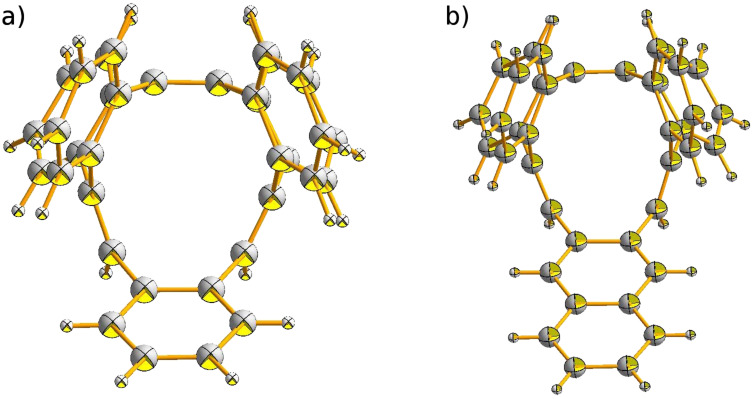
ORTEP drawing of the crystal structures: a) **2** and b) **3**. For additional perspective representations see Supporting Information File 1.

Compounds **2** and **3**, as well as the parent **1**, belong to the class of [12]annulenes and therefore should be antiaromatic according to the Hückel rule. The chemical shifts of the olefinic protons in **2** (6.41 ppm) and **3** (6.49 ppm) are similar to those in **1** (6.45 ppm), which is typical for olefins and do not provide strong evidence for antiaromaticity. The crystal structures of **2** and **3** (Figure 1) confirm the loop-like shape and full conjugation of the compounds, each with *C_S_* symmetry, as is also the case for **1**.

Belt- and tube-like compounds are known to act as π spherands for transition metals such as the Ag^+^ ion [21–22]. After trying several silver salts such as the perchlorate, triflate, hexafluorophosphate and hexafluoroantimonate, silver hexafluoroantimonate gave crystals with **2** on applying the diffusion method with dichloromethane/diethylether (Scheme 2). However, crystals of the corresponding silver complex with **3**, suitable for X-ray structural analysis, could not be obtained.

**Figure 2:**
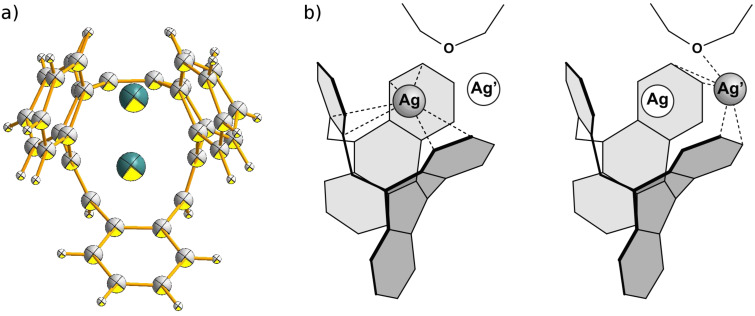
a) ORTEP drawing of the crystal structure of **4**. Ag^+^ and Ag^+^, describe the two disordered positions of Ag^+^. For further perspective views see Supporting Information File 1. b) Simplified structural scheme for the coordination and disorder of the Ag^+^ ion in the X-ray structure of complex **4**.

A number of aromatic hydrocarbons form 1:1-complexes with silver(I). In *all*-*Z*-tribenzo[12]annulene [23], for instance, the Ag^+^ ion is coordinated by three double bonds in a crown-like geometry. Even although three double bonds with a similar geometry are available in our [12]annulene **2**, coordination of the Ag^+^ ion is restricted to the benzene rings. The position of the Ag^+^ ion in the unit cell is disordered over two different coordination sites. The Ag^+^ ion is either coordinated to three formal double bonds of adjacent phenyl rings (Figure 2b, left), or to two formal double bonds and the ether oxygen (Figure 2b, right). The olefinic double bonds are not involved.

The central olefinic double bond in **1** is not activated by electron withdrawing substituents and therefore was reacted with an electron-poor diene in a Diels–Alder reaction with inverse electron demand.

2,3,4,5-Tetrachlorothiophene-1,1-dioxide (**10**) is one of the most electron deficient dienes used in Diels–Alder reactions and is readily accessible by the reaction of tetrachlorothiophene with peroxytrifluoroacetic acid [24–25].

**Scheme 3:**
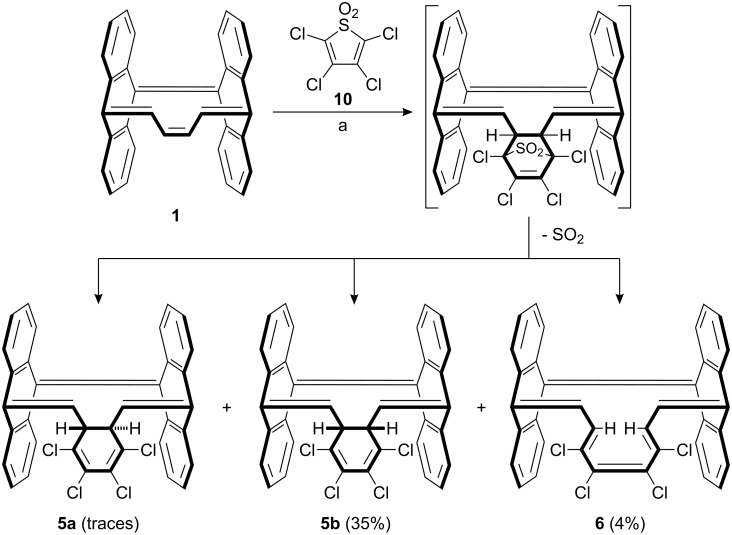
Synthesis of **5** and **6**. (a) Toluene, 90 °C, 15 h.

The Diels–Alder reaction of **1** with an excess of diene **10**, followed by instantaneous elimination of sulfur dioxide furnished compounds **5b** and **6** in 35% and 4% yield, respectively (Scheme 3) along with dimerization products of diene **10**.

Compound **6** is formed by a Woodward–Hoffmann symmetry allowed disrotatory electrocyclic ring opening of **5b**.

Compound **6** is formally a [16]annulene and therefore should be antiaromatic. However, the olefinic *cis* double bond forms dihedral angles of 69° and 79° (X-ray) with the two neighboring *trans* double bonds, and thus is twisted out of conjugation. Therefore, its antiaromatic character should be very small.

**Figure 3:**
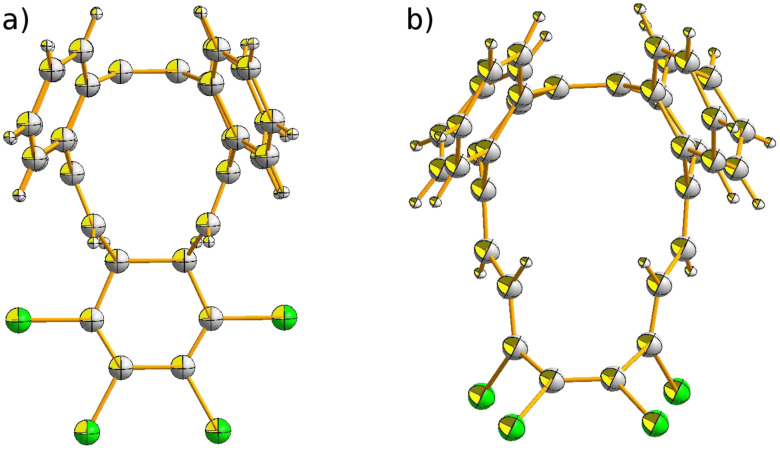
ORTEP drawing of the crystal structures: a) **5b** and b) **6**. For additional perspective representations see Supporting Information File 1.

The *C_S_*-symmetrical compound **5b** and the approximately *C_S_*-symmetrical compound **6** were unambiguously identified from NMR spectral data and crystal structure determination (Figure 3). The X-ray structure analysis of compound **5** confirms that of the two conceivable isomers with *C*_2_ **5a** and *C_S_* symmetry **5b**, the latter was formed as the main product.

On applying the Diels–Alder reaction to compounds **1** and **7** using pyridazine, 1,2-bis(dibromomethyl)benzene and 2,3,4,5-tetrachlorothiophene-1,1-dioxide as dienes the loop-like molecules **2**, **3**, **5b**, and **6** were obtained. All structures were verified by X-ray crystal structure analysis.

## Supporting Information

Supporting information includes experimental procedures and characterization data of all new compounds (**2**, **3**, **4**, **5b** and **6**).

File 1 Procedures and characterization data of compounds **2**, **3**, **4**, **5b** and **6**.
