# Supplementary material for: Novel loop-like aromatic compounds: a further step on the road to nanobelts and nanotubes
Source: Beilstein J Org Chem. 2010 Mar 29;6:30. doi: 10.3762/bjoc.6.30 (PMC2874333; doi:10.3762/bjoc.6.30)
Supplement: File 1 — Procedures and characterization data of compounds 2, 3, 4, 5b and 6. [file Beilstein_J_Org_Chem-06-30-s001.pdf]

Supporting Information
for

Novel loop-like aromatic compounds: a further step on the road to nanobelts and nanotubes

Venkataramana Rajuri[1], Dariush Ajami[2], Gaston R. Schaller[1], Christian Näther[3] and Rainer Herges*[1]

[1]Otto-Diels-Institut für Organische Chemie, Christian-Albrechts-Universität Kiel, Otto-Hahn-Platz 4, 24098 Kiel, Germany, [2]Otto-Diels-Institut für Organische Chemie, current address: The Skaggs Institute for Chemical Biology and Department of Chemistry, The Scripps Research Institute, 10550 North Torrey Pines Road, La Jolla, CA 92037, U.S and [3]Institut für Anorganische Chemie, Christian-Albrechts-Universität Kiel, Otto-Hahn-Platz 6/7, 24098 Kiel, Germany

Email: Rainer Herges* - rherges@oc.uni-kiel.de; Christian Näther - cnaether@ac.uni-kiel.de

* Corresponding author

Procedures and characterization data of compounds 2, 3, 4, 5b and 6.

Table of Contents

1 General Methods and Instruments 3
2 Procedures and Characterization 6
2.1 Compound 2 6
2.2 Compound 3 7
2.3 Compound 4 8
2.4 Compounds 5b and 6 9
3 X-Ray structures 11
3.1 Compound 2 11
3.1.1 Sideview 11
3.1.2 Frontview 11
3.1.3 Topview 11
3.1.4 Data sheets 12
3.2 Compound 3 18
3.2.1 Sideview 18
3.2.2 Frontview 18
3.2.3 Topview 18
3.2.4 Data sheets 19
3.3 Compound 4 25
3.3.1 Sideview 25
3.3.2 Frontview 25
3.3.3 Topview 25
3.3.4 Data sheets 26
3.4 Compound 5b 39
3.4.1 Sideview 39
3.4.2 Frontview 39
3.4.3 Topview 39
3.4.4 Data sheets 40
3.5 Compound 6 46
3.5.1 Sideview 46
3.5.2 Frontview 46
3.5.3 Topview 46
3.5.4 Data sheets 47

1 General Methods and Instruments

General details

All reactions involving air or moisture sensitive reagents or intermediates were carried out under a nitrogen atmosphere in flame-dried glassware.

Purification of solvents

The solvents were purified and dried using common methods, unless used for extraction or column chromatography.
THF and toluene were distilled from sodium, and DMF from CaH_2.

Reagents

Unless otherwise noted, all chemicals were obtained from commercial sources and used as received without further purification.

Thin layer chromatography (TLC)

Thin layer chromatography was performed on Merck silica gel 60 F_{254}.

Column chromatography

Merck Silica gel 60 (0.040 – 0.063 mm) was used for column chromatography (diameter of used column cm × filling height of silica gel cm).

Analytical high performance liquid chromatography (HPLC)

Analytical HPLC was performed on an Agilent system (serial 1100 / 1200) including degasser, quaternary pump, automatic injector, automatic sampler, diode array detector and automatic fraction collector. Designated retention times are not calibrated. The separation was carried out by using one of the following columns from MZ-Analysetechnik including a 20 × 4 mm pre-column:
Kromasil 100 C18 10 µm, 250 × 10 mm
Kromasil 100 C18 10 µm, 250 × 8 mm
Kromasil 100 C18 10 µm, 250 × 4 mm
Kromasil 100 Sil 10 µm, 250 × 8 mm
Kromasil 100 Sil 10 µm, 250 × 4 mm

LiChrospher 60 Si 10 µm, 250 × 4 mm

LiChrospher 60 Si 5 µm, 250 × 8 mm

LiChrospher 100 RP-18 10 µm, 250 × 4 mm

Preparative high performance liquid chromatography (HPLC)

Preparative HPLC was performed on a Gilson system including M305 major pump, M306 mixing pump, 50 SC head pump and Gilson 117 UV detector. Designated retention times are not calibrated. The separation was carried out by using one of the following columns from MZ-Analysetechnik:

Kromasil 100 C18 10 µm, 250 × 20 mm

Silicagel Si 100 12 µm, 250 × 20 mm

Nuclear magnetic resonance spectroscopy (NMR)

NMR spectra were recorded on a Bruker AC–200, Bruker ARX–300, DRX–500 or AV–600 spectrometers. Chemical shifts (δ) are given in ppm.

^{1}H-NMR spectra were calibrated with respect to the following proton signals: 7.26 ($CHCl_3$) and 0.00 ($Si(CH_3)_4$).

^{13}C-NMR spectra were calibrated with reference to the following carbon signals: 77.00 ($CDCl_3$).

Proton and carbon signals were not explicitly assigned.

s = singlet, d = doublet, dd = doublet of doublets, dt = doublet of triplets, t = triplet, tt = triplet of triplets, q = quartet, m = multiplet

Mass spectrometry

According to requirements, mass spectroscopy was recorded on one of the following spectrometers:

MAT 8200 from Finnigan, MarinerTM Biospectrometry Workstation from Applied Biosystems or MALDI–TOF–Spectrometer BiflexTM III from Bruker.

Melting point (MP)

Melting points were determined in capillary tubes using a Büchi Melting Point B-540 and are not corrected.

X-Ray diffraction

X-Ray structures were measured on a Image Plate Diffraction System (IPDS) from STOE & CIE using molybdenum-K_α radiation (wavelength 71.073 pm) at the institute of Inorganic Chemistry at Christian-Albrechts-University Kiel. The analysis of the data was done with the program SHELXS-97.

2 Procedures and Characterization

2.1 Compound 2

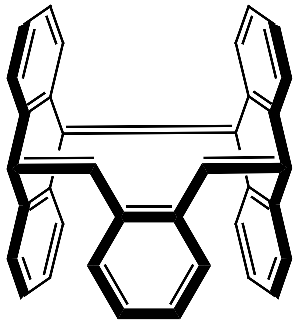

In a flask 9,9',10,10'-tetradehydrodianthracene (**7**) (TDDA) (200 mg, 0.57 mmol, 1 eq), 1,2-bis(dibromomethyl)benzene (**8**) (424 mg, 1 mmol, 1.77 eq) and NaI (2.04 g, 13.6 mmol, 24 eq) were dissolved in dry DMF (10 mL). The mixture was stirred at 60 – 70 °C for 24 h. Afterwards, the reaction mixture was poured into water (150 mL) containing $NaHSO_3$ (2 g). The brown precipitate was purified by column chromatography on silica gel (3 × 25) eluting with hexane / dichloromethane (4 : 1) to give compound **2** as white powder.

Yield:

155 mg (0.34 mmol, 60 %) of **2**

^{1}H-NMR (500 MHz, $CDCl_3$ / TMS, 300 K):

δ = 7.61 – 7.63 (m, 2H), 7.42 – 7.44 (m, 2H), 7.39 – 7.40 (m, 2H), 7,28 – 7,30 (m, 2H), 7.21 – 7.23 (m, 2H), 7.09 – 7.16 (m, 6H), 6.91 (dt, *J* = 7.5, 1.3 Hz, 2H), 6.83 (dt, *J* = 7.5, 1.3 Hz, 2H), 6.41 (s, 2H).

^{13}C-NMR (125 MHz, $CDCl_3$ / TMS, 300 K):

δ = 141.82 (2C), 141.06 (2C), 140.42 (2C), 139.92 (2C), 137. 48 (2C), 137.05 (2C), 135.27 (2C), 129.20 (2CH), 129.02 (2CH), 126.67 (2CH), 126.66 (2CH), 126.54 (2CH), 126.39 (2CH), 126.06 (2CH), 125.47 (2CH), 125.30 (2CH), 125.11 (2CH), 121.89 (2CH).

MS (EI, 70 eV):

m/z (%) = 454 (100) [M^{+}]

MP:

335 °C

2.2 Compound 3

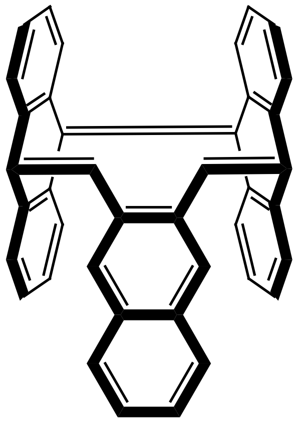

In a flask compound **1** (90 mg, 0.22 mmol, 1 eq), 1,2-bis(dibromomethyl)benzene (**8**) (188 mg, 0.45 mmol, 2 eq) and NaI (0.4 g, 2.7 mmol, 12 eq) were dissolved in dry DMF (1.5 mL). The mixture was stirred at 60 – 70 °C for 15 h. Afterwards, the reaction mixture was poured into water (25 mL) containing $NaHSO_3$ (0.4 g). The brown precipitate was first purified by column chromatography on silica gel (1 × 5) eluting with dichloromethane and finally purified by reversed phase preparative HPLC on kromasil eluting with acetonitrile / water (85 : 15) to give compound **3**.

Yield:

45 mg (89 μmol, 40 %) of **3**

^{1}H-NMR (500 MHz, $CDCl_3$ / TMS, 300 K):

δ = 7.85 – 7.86 (m, 4H), 7.63 – 7.64 (m, 2H), 7.47 – 7.49 (m, 2H), 7.38 – 7.40 (m, 2H), 7.32 – 7.34 (m, 2H), 7.22 – 7.24 (m, 2H), 7.11 – 7.18 (m, 4H), 6.87 (dt, J = 7.5, 1.2 Hz, 2H), 6.79 (dt, J = 7.5, 1.2 Hz, 2H), 6.53 – 6.54 (m, 2H).

^{13}C-NMR (125 MHz, $CDCl_3$ / TMS, 300 K):

δ = 142.07 (2C), 141.21 (2C), 140.51 (2C), 139.96 (2C), 137.20 (2C), 135.76 (2C), 135.25 (2C), 132.73 (2C), 131.22 (2CH), 128.84 (2CH), 127.92 (2CH), 127.74 (2CH), 126.94 (2CH), 126.82 (2CH), 126.73 (4CH), 126.48 (2CH), 125.95 (2CH), 125.62 (2CH), 122.43 (2CH).

MS (EI, 70 eV):

m/z (%) = 504 (100) [M^+]

MP:

333 °C

2.3 Compound 4

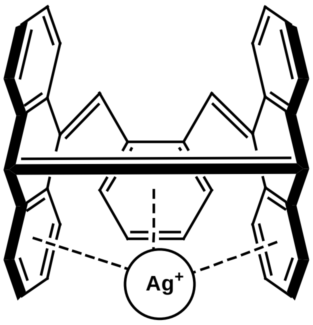

In a flask, under a N_2-atmosphere, compound **2** (15 mg, 44 µmol, 1 eq) was dissolved in dry THF (5 mL) before $AgSbF_6$ (34 mg, 99 µmol, 3 eq) was added. The mixture was stirred at room temperature for 1 h. After removing the solvent in vacuo, the residue was dissolved in a small amount of dichloromethane before diethylether was diffused into the flask to give compound **4** as colorless crystals

Yield:

Not determined

MS (ESI):

m/z (%) = 561 (100) [$(M + Ag)^+$]

2.4 Compounds 5b and 6

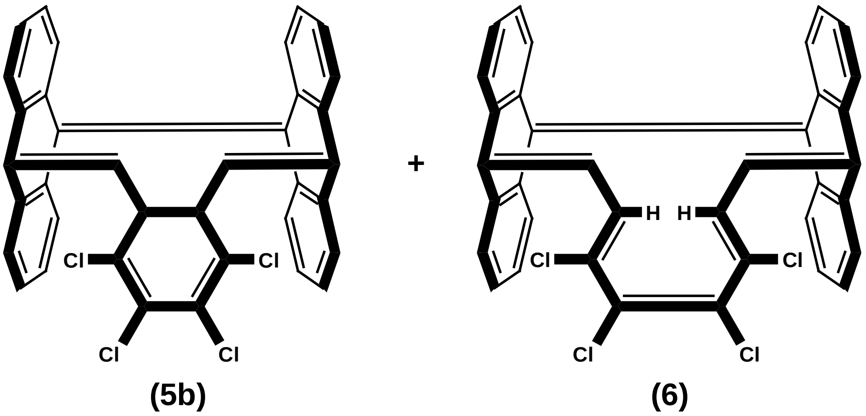

In a flask compound **1** (176 mg, 0.44 mmol, 1 eq) and 1,2,3,4-tetrachlorothiophen-1,1-dioxide (**10**) (276 mg, 1.1 mmol, 2.5 eq) were dissolved in toluene (15 mL). The mixture was stirred at 90 °C for 15 h. After removing the solvent in vacuo, the crude products were dissolved in dichloromethane and first purified by column chromatography on silica gel (1 × 5) eluting with dichloromethane. Further purification by reversed phase preparative HPLC on kromasil eluting with acetonitrile / water (85 : 15) gave pure compound **5b** and impure compound **6**, which was finally purified by normal phase preparative HPLC on silica gel eluting with heptane to give pure compound **6**.

Yields:

90 mg (0.15 mmol, 35 %) of **5b**

10 mg (17 µmol, 4 %) of **6**

Characterization of 5b

^{1}H-NMR (600 MHz, $CDCl_3$ / TMS, 300 K):

δ = 7.56 – 7.57 (m, 2H), 7.53 – 7.55 (m, 2H), 7.37 – 7.38 (m, 2H), 7.17 – 7.18 (m, 2H), 7.08 – 7.13 (m, 4H), 7.02 – 7.06 (m, 4H), 5.51 – 5.53 (m, 2H), 4.32 – 4,34 (m, 2H).

^{13}C-NMR (150 MHz, $CDCl_3$ / TMS, 300 K):

δ = 141.60 (2C), 140.70 (2C), 140.67 (2C), 139.48 (2C), 137.50 (2C), 134.49 (2C), 131.06 (2C), 127.37 (2CH), 126.69 (2CH), 126.38 (4CH), 126.10 (2CH), 126.04 (2CH), 125.61 (2CH), 125.08 (2CH), 123.65 (2C), 122.21 (2CH), 45.13 (2CH).

MS (EI, 70 eV):

m/z (%) = 594 (59) [M^+], 559 (100) [$(M – Cl)^+$]

MP:

319 °C

Characterization of 6

^{1}H-NMR (500 MHz, $CDCl_3$ / TMS, 300 K):

δ = 7.77 – 7.78 (m, 2H), 7.73 – 7.75 (m, 2H), 7.36 – 7.38 (m, 2H), 7.09 – 7.22 (m, 10H), 6.58 (d, *J* = 10.5 Hz, 2H), 6.32 (d, *J* = 10.5 Hz, 2H).

^{13}C-NMR (125 MHz, $CDCl_3$ / TMS, 300 K):

δ = 144.73 (2C), 138.92 (2C), 138.38 (2C), 138.30 (2C), 135.45 (2C), 134.45 (2C), 133.86 (2C), 133.28 (2CH), 128.53 (2CH), 127.86 (2CH), 127.24 (2CH), 127.08 (2CH), 126.51 (2CH), 126.32 (2CH), 125.92 (2CH, 2C), 122.59 (2CH), 120.34 (2CH).

MS (EI, 70 eV):

m/z (%) = 594 (53) [M^+], 559 (97) [$(M – Cl)^+$]

MP:

319 °C

3 X-Ray crystal structures

3.1 Compound 2

3.1.1 Sideview

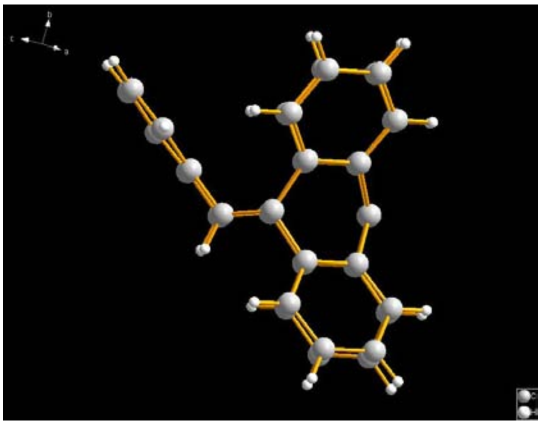

3.1.2 Frontview

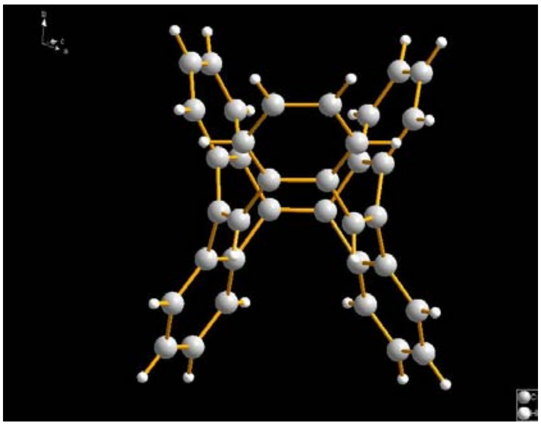

3.1.3 Topview

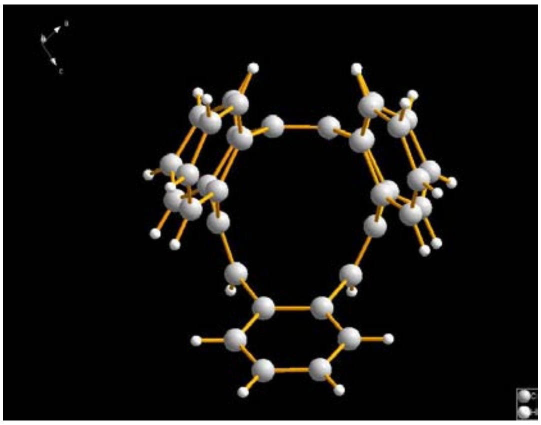

3.1.4 Data sheets

Table S1: Crystal data and structure refinement for compound **2** (herges32).

Identification code	herges32	
Empirical formula	$C_{36}H_{22}$	
Formula weight	454.54	
Temperature	170(2) K	
Wavelength	0.71073 Å	
Crystal system	monoclinic	
Space group	$P2_1/n$	
Unit cell dimensions	a = 9.0130(6) Å	α = 90°.
	b = 16.8487(13) Å	β = 102.871(8)°.
	c = 16.1977(12) Å	γ = 90°.
Volume	2397.9(3) Å^3	
Z	4	
Density (calculated)	1.259 Mg/m^3	
Absorption coefficient	0.071 mm^{-1}	
F(000)	952	
Crystal size	0.3 x 0.2 x 0.2 mm^3	
Theta range for data collection	2.61 to 26.02°.	
Index ranges	-10<=h<=11, -20<=k<=20, -13<=l<=19	
Reflections collected	11980	
Independent reflections	4487 [R(int) = 0.0433]	
Completeness to theta = 26.02°	94.9 %	
Refinement method	Full-matrix least-squares on F^2	
Data / restraints / parameters	4487 / 0 / 326	
Goodness-of-fit on F^2	1.009	
Final R indices [I>2sigma(I)]	R1 = 0.0429, wR2 = 0.1073	
R indices (all data)	R1 = 0.0615, wR2 = 0.1168	
Extinction coefficient	0.017(4)	
Largest diff. peak and hole	0.788 and -0.197 e.Å^{-3}	

Comments:

All non-hydrogen atoms were refined anisotropically. The C-H hydrogen atoms were positioned with idealized geometry and refined using a riding model.

Table S2: Atomic coordinates (× 10^4) and equivalent isotropic displacement parameters ($Å^2$ × 10^3). U(eq) is defined as one third of the trace of the orthogonalized U_{ij} tensor.

	x	y	z	U(eq)
C(1)	1811(2)	6615(1)	3981(1)	26(1)
C(2)	516(2)	7023(1)	4077(1)	32(1)
C(3)	537(2)	7500(1)	4774(1)	35(1)
C(4)	1865(2)	7577(1)	5390(1)	36(1)
C(5)	3166(2)	7166(1)	5314(1)	33(1)
C(6)	3165(2)	6683(1)	4619(1)	26(1)
C(7)	1869(2)	6103(1)	3239(1)	26(1)
C(8)	4507(2)	6208(1)	4510(1)	26(1)
C(11)	4783(2)	6703(1)	2077(1)	21(1)
C(12)	4085(2)	5939(1)	1715(1)	22(1)
C(13)	4698(2)	5411(1)	1220(1)	28(1)
C(14)	3932(2)	4706(1)	938(1)	33(1)
C(15)	2556(2)	4533(1)	1140(1)	36(1)
C(16)	1928(2)	5055(1)	1636(1)	32(1)
C(17)	2683(2)	5760(1)	1926(1)	25(1)
C(18)	2160(2)	6339(1)	2501(1)	25(1)
C(19)	2318(2)	7178(1)	2243(1)	24(1)
C(20)	1222(2)	7766(1)	2231(1)	31(1)
C(21)	1417(2)	8516(1)	1913(1)	35(1)
C(22)	2689(2)	8676(1)	1595(1)	33(1)
C(23)	3807(2)	8105(1)	1616(1)	27(1)
C(24)	3639(2)	7361(1)	1953(1)	22(1)
C(31)	6074(2)	6753(1)	2701(1)	21(1)
C(32)	6939(2)	6046(1)	3114(1)	21(1)
C(33)	7883(2)	5560(1)	2762(1)	25(1)
C(34)	8635(2)	4924(1)	3228(1)	28(1)
C(35)	8444(2)	4770(1)	4036(1)	30(1)
C(36)	7511(2)	5260(1)	4398(1)	27(1)
C(37)	6765(2)	5900(1)	3942(1)	22(1)
C(38)	5741(2)	6466(1)	4263(1)	22(1)
C(39)	6099(2)	7307(1)	4092(1)	22(1)
C(40)	6267(2)	7917(1)	4689(1)	28(1)
C(41)	6719(2)	8667(1)	4485(1)	33(1)
C(42)	7026(2)	8806(1)	3699(1)	33(1)
C(43)	6852(2)	8203(1)	3092(1)	27(1)
C(44)	6364(2)	7456(1)	3283(1)	21(1)

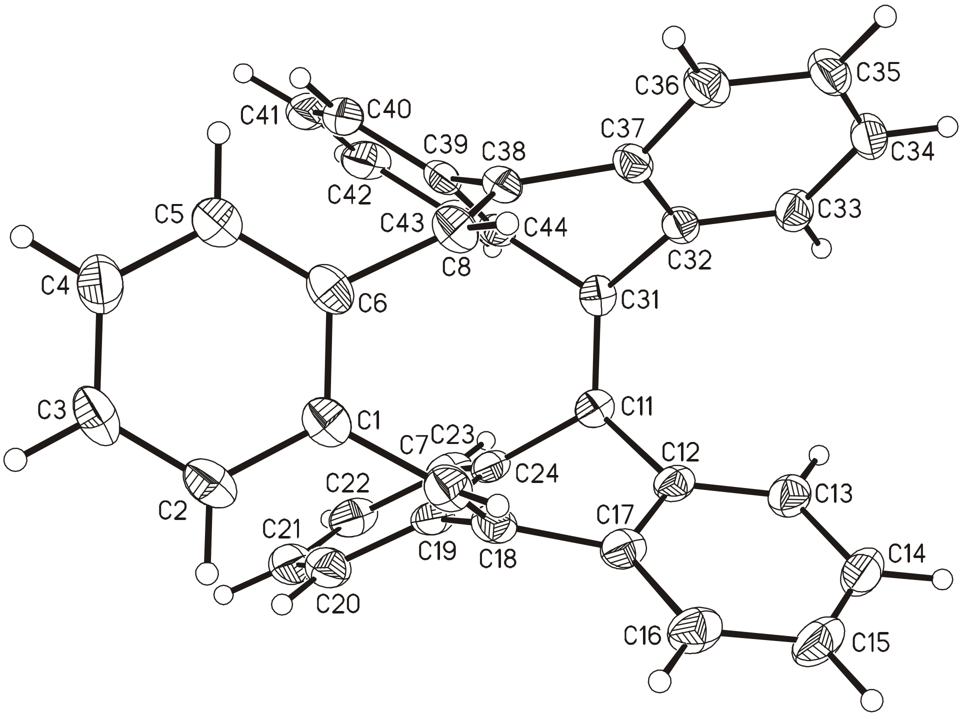

Table S3: Bond lengths [Å].

C(1)-C(2)	1.393(2)	C(19)-C(24)	1.408(2)
C(1)-C(6)	1.417(2)	C(20)-C(21)	1.390(2)
C(1)-C(7)	1.489(2)	C(21)-C(22)	1.384(3)
C(2)-C(3)	1.383(3)	C(22)-C(23)	1.388(2)
C(3)-C(4)	1.383(3)	C(23)-C(24)	1.390(2)
C(4)-C(5)	1.391(2)	C(31)-C(32)	1.497(2)
C(5)-C(6)	1.389(2)	C(31)-C(44)	1.501(2)
C(6)-C(8)	1.493(2)	C(32)-C(33)	1.391(2)
C(7)-C(18)	1.339(2)	C(32)-C(37)	1.408(2)
C(8)-C(38)	1.336(2)	C(33)-C(34)	1.395(2)
C(11)-C(31)	1.363(2)	C(34)-C(35)	1.383(3)
C(11)-C(12)	1.4948(19)	C(35)-C(36)	1.396(2)
C(11)-C(24)	1.496(2)	C(36)-C(37)	1.392(2)
C(12)-C(13)	1.392(2)	C(37)-C(38)	1.498(2)
C(12)-C(17)	1.413(2)	C(38)-C(39)	1.492(2)
C(13)-C(14)	1.399(2)	C(39)-C(40)	1.398(2)
C(14)-C(15)	1.382(3)	C(39)-C(44)	1.405(2)
C(15)-C(16)	1.394(3)	C(40)-C(41)	1.390(2)
C(16)-C(17)	1.397(2)	C(41)-C(42)	1.382(3)
C(17)-C(18)	1.495(2)	C(42)-C(43)	1.397(2)
C(18)-C(19)	1.491(2)	C(43)-C(44)	1.390(2)
C(19)-C(20)	1.396(2)		

Table S4: Bond angles [°].

C(2)-C(1)-C(6)	119.00(16)	C(22)-C(21)-C(20)	119.93(15)
C(2)-C(1)-C(7)	123.85(16)	C(21)-C(22)-C(23)	120.90(15)
C(6)-C(1)-C(7)	117.15(14)	C(22)-C(23)-C(24)	119.49(16)
C(3)-C(2)-C(1)	121.27(16)	C(23)-C(24)-C(19)	120.17(14)
C(2)-C(3)-C(4)	119.76(16)	C(23)-C(24)-C(11)	126.05(15)
C(3)-C(4)-C(5)	119.97(17)	C(19)-C(24)-C(11)	113.75(12)
C(6)-C(5)-C(4)	121.07(17)	C(11)-C(31)-C(32)	123.77(12)
C(5)-C(6)-C(1)	118.92(15)	C(11)-C(31)-C(44)	120.97(13)
C(5)-C(6)-C(8)	123.73(15)	C(32)-C(31)-C(44)	110.30(13)
C(1)-C(6)-C(8)	117.33(15)	C(33)-C(32)-C(37)	119.69(14)
C(18)-C(7)-C(1)	126.71(14)	C(33)-C(32)-C(31)	126.23(15)
C(38)-C(8)-C(6)	127.69(14)	C(37)-C(32)-C(31)	114.06(13)
C(31)-C(11)-C(12)	123.96(13)	C(32)-C(33)-C(34)	119.95(16)
C(31)-C(11)-C(24)	120.22(13)	C(35)-C(34)-C(33)	120.49(15)
C(12)-C(11)-C(24)	111.56(13)	C(34)-C(35)-C(36)	119.99(15)
C(13)-C(12)-C(17)	119.68(14)	C(37)-C(36)-C(35)	120.04(16)
C(13)-C(12)-C(11)	126.11(15)	C(36)-C(37)-C(32)	119.82(14)
C(17)-C(12)-C(11)	114.21(13)	C(36)-C(37)-C(38)	124.89(15)
C(12)-C(13)-C(14)	119.99(17)	C(32)-C(37)-C(38)	115.29(13)
C(15)-C(14)-C(13)	120.41(16)	C(8)-C(38)-C(39)	126.38(13)
C(14)-C(15)-C(16)	120.23(15)	C(8)-C(38)-C(37)	120.99(13)
C(15)-C(16)-C(17)	120.11(17)	C(39)-C(38)-C(37)	111.40(13)
C(16)-C(17)-C(12)	119.59(15)	C(40)-C(39)-C(44)	119.91(14)
C(16)-C(17)-C(18)	124.54(15)	C(40)-C(39)-C(38)	124.37(15)
C(12)-C(17)-C(18)	115.78(13)	C(44)-C(39)-C(38)	115.60(13)
C(7)-C(18)-C(19)	125.56(15)	C(41)-C(40)-C(39)	119.77(17)
C(7)-C(18)-C(17)	120.95(13)	C(42)-C(41)-C(40)	120.16(15)
C(19)-C(18)-C(17)	112.37(14)	C(41)-C(42)-C(43)	120.71(15)
C(20)-C(19)-C(24)	119.29(14)	C(44)-C(43)-C(42)	119.56(17)
C(20)-C(19)-C(18)	124.42(15)	C(43)-C(44)-C(39)	119.83(14)
C(24)-C(19)-C(18)	116.21(13)	C(43)-C(44)-C(31)	126.47(15)
C(21)-C(20)-C(19)	120.12(17)	C(39)-C(44)-C(31)	113.70(12)

Table S5: Anisotropic displacement parameters ($Å^2 \times 10^3$). The anisotropic displacement factor exponent takes the form: $-2\pi^2[h^2 a^{*2}U_{11} + ... + 2 h k a^* b^* U_{12}]$

	U_{11}	U_{22}	U_{33}	U_{23}	U_{13}	U_{12}
C(1)	22(1)	23(1)	32(1)	5(1)	9(1)	-2(1)
C(2)	20(1)	34(1)	41(1)	3(1)	6(1)	2(1)
C(3)	24(1)	34(1)	49(1)	0(1)	15(1)	7(1)
C(4)	35(1)	36(1)	38(1)	-4(1)	14(1)	6(1)
C(5)	28(1)	37(1)	33(1)	0(1)	5(1)	5(1)
C(6)	21(1)	26(1)	32(1)	6(1)	10(1)	2(1)
C(7)	22(1)	22(1)	35(1)	1(1)	6(1)	-3(1)
C(8)	24(1)	24(1)	30(1)	5(1)	6(1)	4(1)
C(11)	24(1)	19(1)	20(1)	2(1)	5(1)	-2(1)
C(12)	27(1)	18(1)	19(1)	1(1)	-1(1)	-1(1)
C(13)	34(1)	27(1)	23(1)	0(1)	2(1)	3(1)
C(14)	45(1)	25(1)	26(1)	-5(1)	0(1)	3(1)
C(15)	49(1)	21(1)	32(1)	-4(1)	-3(1)	-6(1)
C(16)	34(1)	25(1)	34(1)	0(1)	1(1)	-8(1)
C(17)	28(1)	20(1)	24(1)	3(1)	-1(1)	-1(1)
C(18)	19(1)	23(1)	31(1)	0(1)	2(1)	-2(1)
C(19)	23(1)	22(1)	23(1)	-2(1)	-4(1)	-1(1)
C(20)	27(1)	29(1)	34(1)	-1(1)	-2(1)	4(1)
C(21)	36(1)	25(1)	38(1)	-3(1)	-7(1)	9(1)
C(22)	43(1)	18(1)	31(1)	2(1)	-8(1)	-1(1)
C(23)	34(1)	21(1)	23(1)	1(1)	-4(1)	-5(1)
C(24)	25(1)	19(1)	19(1)	-2(1)	-3(1)	-1(1)
C(31)	22(1)	18(1)	24(1)	1(1)	8(1)	-2(1)
C(32)	16(1)	18(1)	28(1)	-2(1)	3(1)	-2(1)
C(33)	24(1)	23(1)	29(1)	-5(1)	6(1)	-1(1)
C(34)	23(1)	22(1)	39(1)	-6(1)	5(1)	3(1)
C(35)	27(1)	22(1)	38(1)	2(1)	1(1)	6(1)
C(36)	26(1)	24(1)	29(1)	3(1)	4(1)	3(1)
C(37)	17(1)	20(1)	28(1)	-1(1)	3(1)	0(1)
C(38)	19(1)	22(1)	23(1)	0(1)	1(1)	4(1)
C(39)	15(1)	22(1)	27(1)	-1(1)	0(1)	4(1)
C(40)	25(1)	28(1)	27(1)	-4(1)	-1(1)	5(1)
C(41)	31(1)	24(1)	37(1)	-9(1)	-7(1)	2(1)
C(42)	31(1)	18(1)	44(1)	1(1)	-7(1)	-4(1)
C(43)	23(1)	22(1)	33(1)	2(1)	-2(1)	-2(1)
C(44)	15(1)	19(1)	27(1)	-1(1)	-1(1)	1(1)

Table S6: Hydrogen coordinates ($\times 10^4$) and isotropic displacement parameters ($Å^2 \times 10^3$).

	x	y	z	U(eq)
H(2)	-399	6972	3656	38
H(3)	-357	7773	4830	42
H(4)	1889	7911	5866	43
H(5)	4069	7216	5744	39
H(7)	1681	5553	3294	32
H(8)	4481	5657	4631	31
H(13)	5636	5530	1072	34
H(14)	4360	4344	606	40
H(15)	2036	4056	940	43
H(16)	985	4932	1776	38
H(20)	340	7653	2441	38
H(21)	678	8918	1914	42
H(22)	2798	9183	1360	40
H(23)	4680	8223	1400	32
H(33)	8016	5660	2206	30
H(34)	9282	4594	2987	34
H(35)	8949	4332	4346	35
H(36)	7385	5156	4954	32
H(40)	6074	7820	5233	34
H(41)	6816	9085	4887	39
H(42)	7359	9316	3569	40
H(43)	7064	8303	2553	32

3.2 Compound 3

3.2.1 Sideview

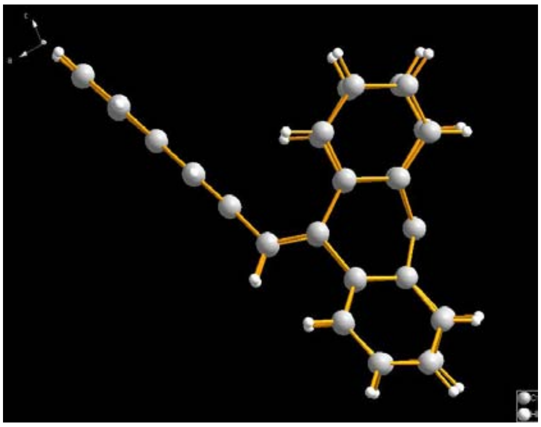

3.2.2 Frontview

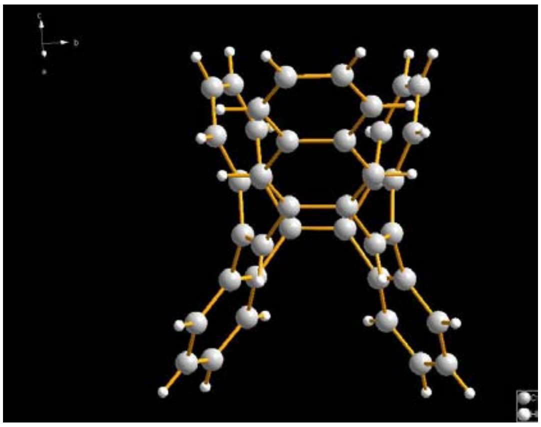

3.2.3 Topview

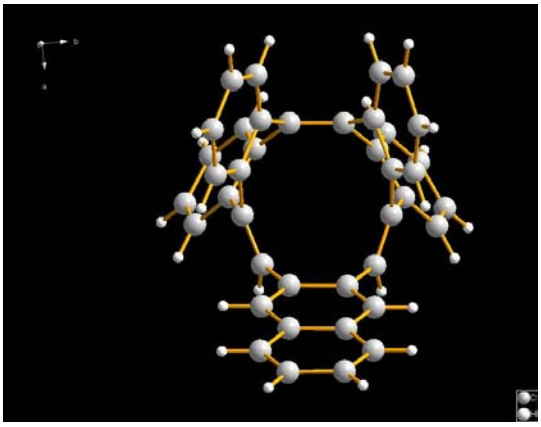

3.2.4 Data sheets

Table S7: Crystal data and structure refinement for compound **3** (herges37).

Identification code	herges37	
Empirical formula	$C_{40}H_{24}$	
Formula weight	504.59	
Temperature	170(2) K	
Wavelength	0.71073 Å	
Crystal system	monoclinic	
Space group	$P2_1/c$	
Unit cell dimensions	a = 9.3217(7) Å	α = 90°.
	b = 14.4237(6) Å	β = 98.185(9)°.
	c = 19.5029(14) Å	γ = 90°.
Volume	2595.5(3) $Å^3$	
Z	4	
Density (calculated)	1.291 Mg/m^3	
Absorption coefficient	0.073 mm^{-1}	
F(000)	1056	
Crystal size	0.4 x 0.3 x 0.3 mm^3	
Theta range for data collection	2.54 to 28.11°.	
Index ranges	-12<=h<=12, -17<=k<=18, -22<=l<=25	
Reflections collected	22114	
Independent reflections	6230 [R(int) = 0.0359]	
Completeness to theta = 28.11°	98.3 %	
Refinement method	Full-matrix least-squares on F^2	
Data / restraints / parameters	6230 / 0 / 362	
Goodness-of-fit on F^2	1.022	
Final R indices [I>2sigma(I)]	R1 = 0.0415, wR2 = 0.1071	
R indices (all data)	R1 = 0.0557, wR2 = 0.1141	
Extinction coefficient	0.022(3)	
Largest diff. peak and hole	0.250 and -0.226 e.$Å^{-3}$	

Comments:

All non-hydrogen atoms were refined anisotropically. The C-H hydrogen atoms were positioned with idealized geometry and refined using a riding model.

Table S8: Atomic coordinates ($\times 10^4$) and equivalent isotropic displacement parameters ($Å^2 \times 10^3$). U(eq) is defined as one third of the trace of the orthogonalized U_{ij} tensor.

	x	y	z	U(eq)
C(1)	6318(1)	3150(1)	2549(1)	24(1)
C(2)	6972(1)	2697(1)	3129(1)	27(1)
C(3)	7485(1)	3185(1)	3748(1)	26(1)
C(4)	8158(1)	2726(1)	4355(1)	34(1)
C(5)	8659(1)	3227(1)	4939(1)	39(1)
C(6)	8532(1)	4195(1)	4944(1)	38(1)
C(7)	7872(1)	4660(1)	4370(1)	33(1)
C(8)	7323(1)	4163(1)	3759(1)	25(1)
C(9)	6644(1)	4621(1)	3154(1)	26(1)
C(10)	6143(1)	4141(1)	2561(1)	23(1)
C(11)	5816(1)	2676(1)	1879(1)	25(1)
C(12)	5494(1)	4596(1)	1901(1)	23(1)
C(21)	4504(1)	2313(1)	1668(1)	22(1)
C(22)	3306(1)	2191(1)	2090(1)	23(1)
C(23)	3467(1)	1797(1)	2747(1)	28(1)
C(24)	2256(2)	1610(1)	3068(1)	33(1)
C(25)	881(2)	1804(1)	2732(1)	36(1)
C(26)	701(1)	2226(1)	2083(1)	32(1)
C(27)	1906(1)	2437(1)	1767(1)	24(1)
C(28)	1921(1)	2989(1)	1121(1)	23(1)
C(29)	2708(1)	2490(1)	616(1)	23(1)
C(30)	2253(1)	2373(1)	-91(1)	29(1)
C(31)	3097(2)	1864(1)	-492(1)	34(1)
C(32)	4404(2)	1485(1)	-193(1)	34(1)
C(33)	4886(1)	1613(1)	507(1)	29(1)
C(34)	4048(1)	2108(1)	917(1)	23(1)
C(41)	4118(1)	4857(1)	1709(1)	21(1)
C(42)	2929(1)	4807(1)	2139(1)	23(1)
C(43)	3010(1)	5189(1)	2800(1)	30(1)
C(44)	1819(2)	5143(1)	3153(1)	39(1)
C(45)	546(2)	4733(1)	2848(1)	38(1)
C(46)	449(1)	4349(1)	2190(1)	31(1)
C(47)	1639(1)	4373(1)	1833(1)	23(1)
C(48)	1776(1)	3922(1)	1153(1)	22(1)
C(49)	2343(1)	4585(1)	670(1)	22(1)
C(50)	1770(1)	4744(1)	-19(1)	27(1)
C(51)	2386(1)	5413(1)	-399(1)	31(1)
C(52)	3594(1)	5909(1)	-104(1)	31(1)
C(53)	4197(1)	5744(1)	581(1)	26(1)
C(54)	3573(1)	5092(1)	971(1)	22(1)

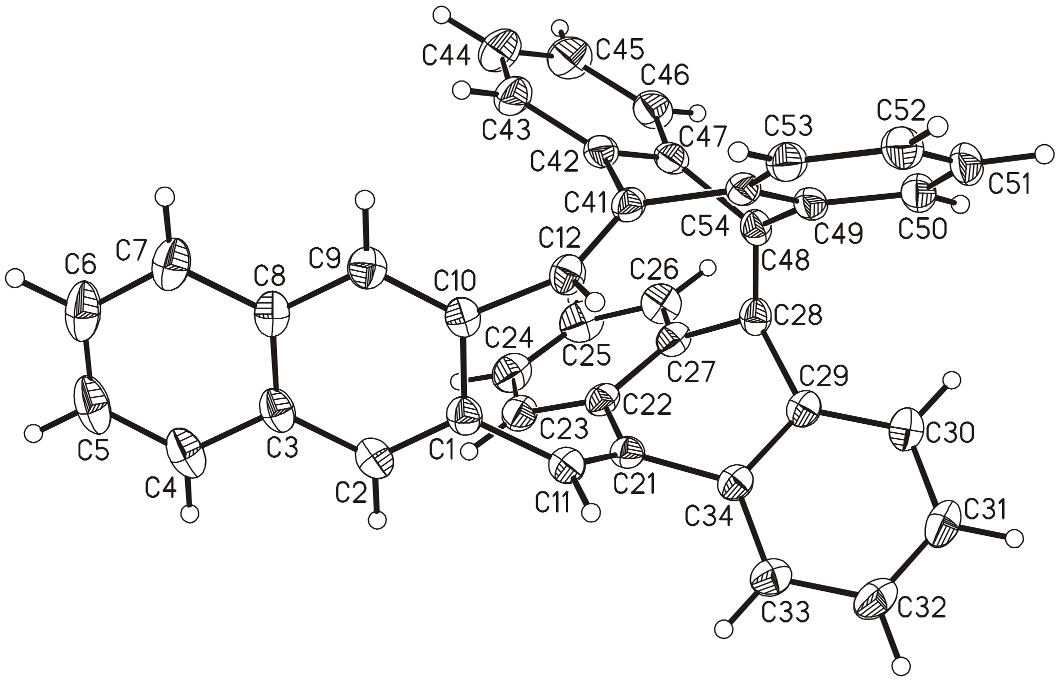

Table S9: Bond lengths [Å].

C(1)-C(2)	1.3713(16)	C(28)-C(48)	1.3547(17)
C(1)-C(10)	1.4402(17)	C(28)-C(29)	1.4940(15)
C(1)-C(11)	1.4900(16)	C(29)-C(30)	1.3936(16)
C(2)-C(3)	1.4217(17)	C(29)-C(34)	1.4139(16)
C(3)-C(8)	1.4185(18)	C(30)-C(31)	1.3942(18)
C(3)-C(4)	1.4216(16)	C(31)-C(32)	1.387(2)
C(4)-C(5)	1.373(2)	C(32)-C(33)	1.3888(18)
C(5)-C(6)	1.402(2)	C(33)-C(34)	1.3908(16)
C(6)-C(7)	1.3732(19)	C(41)-C(42)	1.4836(15)
C(7)-C(8)	1.4217(17)	C(41)-C(54)	1.4941(15)
C(8)-C(9)	1.4198(16)	C(42)-C(43)	1.3940(16)
C(9)-C(10)	1.3712(16)	C(42)-C(47)	1.4107(16)
C(10)-C(12)	1.4932(15)	C(43)-C(44)	1.3891(18)
C(11)-C(21)	1.3403(16)	C(44)-C(45)	1.383(2)
C(12)-C(41)	1.3384(16)	C(45)-C(46)	1.3897(18)
C(21)-C(22)	1.4895(15)	C(46)-C(47)	1.3914(15)
C(21)-C(34)	1.4951(15)	C(47)-C(48)	1.4982(15)
C(22)-C(23)	1.3904(16)	C(48)-C(49)	1.4911(15)
C(22)-C(27)	1.4115(16)	C(49)-C(50)	1.3914(15)
C(23)-C(24)	1.3927(17)	C(49)-C(54)	1.4152(16)
C(24)-C(25)	1.383(2)	C(50)-C(51)	1.3897(18)
C(25)-C(26)	1.3930(18)	C(51)-C(52)	1.388(2)
C(26)-C(27)	1.3890(16)	C(52)-C(53)	1.3935(16)
C(27)-C(28)	1.4927(16)	C(53)-C(54)	1.3893(16)

Table S10: Bond angles [°].

C(2)-C(1)-C(10)	119.82(11)	C(30)-C(29)-C(34)	119.34(11)
C(2)-C(1)-C(11)	123.54(11)	C(30)-C(29)-C(28)	126.96(11)
C(10)-C(1)-C(11)	116.59(10)	C(34)-C(29)-C(28)	113.69(10)
C(1)-C(2)-C(3)	121.46(12)	C(29)-C(30)-C(31)	120.14(12)
C(8)-C(3)-C(4)	119.08(11)	C(32)-C(31)-C(30)	120.25(12)
C(8)-C(3)-C(2)	118.73(10)	C(31)-C(32)-C(33)	120.21(12)
C(4)-C(3)-C(2)	122.19(12)	C(32)-C(33)-C(34)	120.23(12)
C(5)-C(4)-C(3)	120.24(14)	C(33)-C(34)-C(29)	119.79(11)
C(4)-C(5)-C(6)	120.66(12)	C(33)-C(34)-C(21)	123.99(10)
C(7)-C(6)-C(5)	120.65(13)	C(29)-C(34)-C(21)	116.15(10)
C(6)-C(7)-C(8)	120.17(14)	C(12)-C(41)-C(42)	126.21(10)
C(3)-C(8)-C(9)	119.10(10)	C(12)-C(41)-C(54)	120.99(10)
C(3)-C(8)-C(7)	119.16(11)	C(42)-C(41)-C(54)	112.09(9)
C(9)-C(8)-C(7)	121.72(12)	C(43)-C(42)-C(47)	119.79(11)
C(10)-C(9)-C(8)	121.62(11)	C(43)-C(42)-C(41)	123.83(11)
C(9)-C(10)-C(1)	119.27(10)	C(47)-C(42)-C(41)	116.33(10)
C(9)-C(10)-C(12)	123.55(11)	C(44)-C(43)-C(42)	119.92(12)
C(1)-C(10)-C(12)	117.07(10)	C(45)-C(44)-C(43)	120.27(12)
C(21)-C(11)-C(1)	127.39(10)	C(44)-C(45)-C(46)	120.47(12)
C(41)-C(12)-C(10)	128.29(10)	C(45)-C(46)-C(47)	120.05(12)
C(11)-C(21)-C(22)	126.85(10)	C(46)-C(47)-C(42)	119.47(11)
C(11)-C(21)-C(34)	119.85(10)	C(46)-C(47)-C(48)	127.34(11)
C(22)-C(21)-C(34)	112.79(9)	C(42)-C(47)-C(48)	113.05(9)
C(23)-C(22)-C(27)	119.07(11)	C(28)-C(48)-C(49)	124.07(10)
C(23)-C(22)-C(21)	124.54(11)	C(28)-C(48)-C(47)	119.64(10)
C(27)-C(22)-C(21)	116.23(10)	C(49)-C(48)-C(47)	111.32(10)
C(22)-C(23)-C(24)	120.32(12)	C(50)-C(49)-C(54)	119.50(11)
C(25)-C(24)-C(23)	120.29(12)	C(50)-C(49)-C(48)	126.43(11)
C(24)-C(25)-C(26)	120.15(12)	C(54)-C(49)-C(48)	114.06(9)
C(27)-C(26)-C(25)	119.90(12)	C(51)-C(50)-C(49)	119.88(12)
C(26)-C(27)-C(22)	120.10(11)	C(52)-C(51)-C(50)	120.66(11)
C(26)-C(27)-C(28)	126.64(11)	C(51)-C(52)-C(53)	120.07(11)
C(22)-C(27)-C(28)	113.06(10)	C(54)-C(53)-C(52)	119.89(12)
C(48)-C(28)-C(27)	118.46(10)	C(53)-C(54)-C(49)	119.97(10)
C(48)-C(28)-C(29)	124.85(10)	C(53)-C(54)-C(41)	124.72(11)
C(27)-C(28)-C(29)	111.37(10)	C(49)-C(54)-C(41)	115.31(10)

Table S11: Anisotropic displacement parameters ($Å^2 \times 10^3$). The anisotropic displacement factor exponent takes the form: $-2\pi^2[h^2 a^{*2}U_{11} + ... + 2 h k a^* b^* U_{12}]$.

	U_{11}	U_{22}	U_{33}	U_{23}	U_{13}	U_{12}
C(1)	19(1)	28(1)	24(1)	0(1)	2(1)	-1(1)
C(2)	25(1)	28(1)	29(1)	4(1)	1(1)	0(1)
C(3)	18(1)	36(1)	24(1)	6(1)	3(1)	-2(1)
C(4)	25(1)	47(1)	30(1)	12(1)	1(1)	1(1)
C(5)	26(1)	67(1)	23(1)	13(1)	0(1)	-2(1)
C(6)	30(1)	65(1)	20(1)	-2(1)	3(1)	-11(1)
C(7)	30(1)	45(1)	24(1)	-3(1)	5(1)	-9(1)
C(8)	20(1)	36(1)	20(1)	1(1)	4(1)	-5(1)
C(9)	27(1)	26(1)	25(1)	2(1)	4(1)	-2(1)
C(10)	19(1)	28(1)	21(1)	3(1)	4(1)	-1(1)
C(11)	25(1)	26(1)	24(1)	0(1)	3(1)	2(1)
C(12)	26(1)	25(1)	19(1)	1(1)	5(1)	-2(1)
C(21)	26(1)	19(1)	22(1)	0(1)	4(1)	2(1)
C(22)	28(1)	18(1)	22(1)	-2(1)	5(1)	-2(1)
C(23)	36(1)	22(1)	24(1)	1(1)	4(1)	0(1)
C(24)	46(1)	28(1)	27(1)	4(1)	12(1)	-2(1)
C(25)	39(1)	34(1)	37(1)	4(1)	18(1)	-6(1)
C(26)	28(1)	33(1)	35(1)	2(1)	8(1)	-4(1)
C(27)	27(1)	23(1)	23(1)	-2(1)	6(1)	-2(1)
C(28)	19(1)	29(1)	21(1)	0(1)	2(1)	-2(1)
C(29)	24(1)	23(1)	22(1)	-1(1)	4(1)	-3(1)
C(30)	30(1)	33(1)	23(1)	-1(1)	1(1)	-4(1)
C(31)	44(1)	37(1)	21(1)	-4(1)	6(1)	-5(1)
C(32)	41(1)	35(1)	28(1)	-6(1)	14(1)	0(1)
C(33)	30(1)	29(1)	29(1)	-2(1)	8(1)	1(1)
C(34)	25(1)	21(1)	23(1)	-1(1)	5(1)	-3(1)
C(41)	28(1)	19(1)	18(1)	0(1)	5(1)	0(1)
C(42)	28(1)	21(1)	19(1)	3(1)	6(1)	5(1)
C(43)	38(1)	30(1)	22(1)	-2(1)	7(1)	3(1)
C(44)	49(1)	47(1)	25(1)	-5(1)	15(1)	8(1)
C(45)	38(1)	48(1)	31(1)	2(1)	19(1)	9(1)
C(46)	27(1)	37(1)	29(1)	4(1)	9(1)	6(1)
C(47)	25(1)	24(1)	21(1)	4(1)	6(1)	7(1)
C(48)	18(1)	29(1)	19(1)	2(1)	2(1)	3(1)
C(49)	22(1)	26(1)	19(1)	2(1)	5(1)	7(1)
C(50)	25(1)	36(1)	21(1)	0(1)	3(1)	8(1)
C(51)	35(1)	40(1)	17(1)	6(1)	5(1)	13(1)
C(52)	43(1)	29(1)	23(1)	7(1)	11(1)	7(1)
C(53)	34(1)	24(1)	23(1)	2(1)	8(1)	3(1)
C(54)	26(1)	22(1)	18(1)	1(1)	6(1)	6(1)

Table S12: Hydrogen coordinates ($\times 10^4$) and isotropic displacement parameters ($Å^2 \times 10^3$).

	x	y	z	U(eq)
H(2)	7084	2043	3116	33
H(4)	8261	2071	4357	41
H(5)	9096	2913	5344	47
H(6)	8907	4533	5348	46
H(7)	7782	5316	4380	39
H(9)	6535	5275	3160	31
H(11)	6505	2626	1565	30
H(12)	6141	4716	1577	28
H(23)	4408	1655	2979	33
H(24)	2375	1348	3519	40
H(25)	56	1649	2944	43
H(26)	-243	2369	1856	38
H(30)	1366	2641	-300	35
H(31)	2774	1777	-972	41
H(32)	4972	1136	-469	40
H(33)	5791	1361	708	34
H(43)	3878	5480	3008	36
H(44)	1880	5395	3606	47
H(45)	-269	4714	3090	46
H(46)	-431	4070	1983	37
H(50)	960	4395	-228	33
H(51)	1976	5532	-864	37
H(52)	4011	6361	-370	37
H(53)	5033	6077	780	32

3.3 Compound 4

3.3.1 Sideview

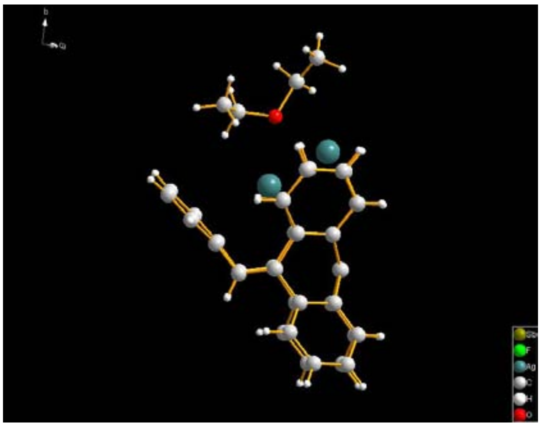

3.3.2 Frontview

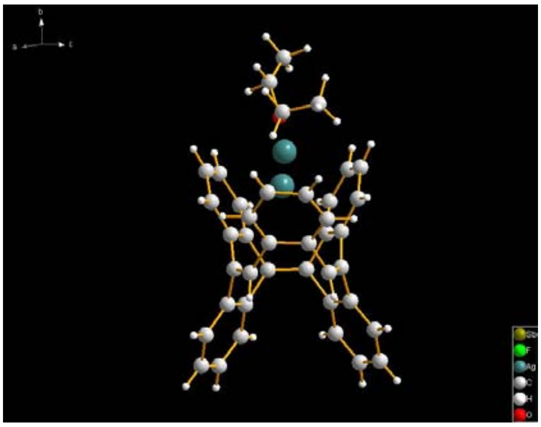

3.3.3 Topview

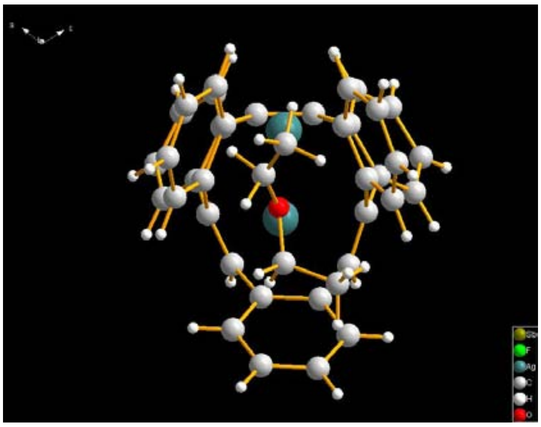

3.3.4 Data sheets

Table S13: Crystal data and structure refinement for compound **4** (herge39a).

Identification code	herge39a	
Empirical formula	$C_{40}H_{32}AgF_6OSb$	
Formula weight	872.28	
Temperature	170(2) K	
Wavelength	0.71073 Å	
Crystal system	monoclinic	
Space group	Cc	
Unit cell dimensions	a = 17.7185(14) Å	α = 90°.
	b = 23.6763(16) Å	β = 108.196(9)°.
	c = 16.7757(13) Å	γ = 90°.
Volume	6685.6(9) $Å^3$	
Z	8	
Density (calculated)	1.733 Mg/m^3	
Absorption coefficient	1.460 mm^{-1}	
F(000)	3456	
Crystal size	0.2 x 0.15 x 0.08 mm^3	
Theta range for data collection	2.56 to 26.02°.	
Index ranges	-21<=h<=21, -29<=k<=29, -20<=l<=20	
Reflections collected	24672	
Independent reflections	12620 [R(int) = 0.0547]	
Completeness to theta = 26.02°	99.2 %	
Refinement method	Full-matrix least-squares on F^2	
Data / restraints / parameters	12620 / 46 / 871	
Goodness-of-fit on F^2	1.080	
Final R indices [I>2sigma(I)]	R1 = 0.0691, wR2 = 0.1684	
R indices (all data)	R1 = 0.0968, wR2 = 0.1904	
Absolute structure parameter	0.00(3)	
Extinction coefficient	0.00145(19)	
Largest diff. peak and hole	1.223 and -1.252 e.$Å^{-3}$	

Comments:

All non-hydrogen atoms and with the exception of one fluoro atom were refined anisotropically. The H atoms were positioned with idealized geometry and refined using a riding model. A numerical absorption correction was performed Tmin./max= 0.5840 / 0.8766. There are two crystallographically independent comlexes in the asymmetric unit. Each of the

two silver atoms is disordered over two positions and was refined using a split model. Some of the fluoro atoms of both hexafluoroantimonate anions exhibit unusual high anisotropic displacement parameters indicating disorder. One of these fluoro atoms could only be refined isotropically. Analysis of the structure shows that both crystallogrphically independent complexes can be transferred into each other by half a translation into the direction of the a-axis. However, if the structure is refined in the smaller cell, disorder and unusual short bond distances between carbon and fluoro atoms occurs.

Table S14: Atomic coordinates (× 10^4) and equivalent isotropic displacement parameters ($Å^2 \times 10^3$). U(eq) is defined as one third of the trace of the orthogonalized U_{ij} tensor.

	x	y	z	U(eq)
Sb(1)	6699(1)	9378(1)	5395(1)	55(1)
F(1)	6696(6)	8586(3)	5372(6)	85(2)
F(2)	6712(7)	10178(3)	5408(6)	91(3)
F(3)	6707(9)	9394(4)	4310(5)	113(4)
F(4)	7841(5)	9368(3)	5729(7)	95(3)
F(5)	6839(8)	9357(4)	6537(6)	113(4)
F(6)	5705(7)	9377(6)	5227(13)	156(6)
Sb(2)	11589(1)	9382(1)	5321(1)	76(1)
F(11)	11681(7)	8612(3)	5274(7)	134(5)
F(12)	11613(7)	10169(4)	5335(6)	134(5)
F(13)	12753(6)	9394(4)	5658(8)	133(5)
F(14)	11811(7)	9333(5)	6452(6)	166(7)
F(15)	11736(9)	9394(4)	4261(7)	159(7)
F(16)	10544(7)	9330(6)	5128(10)	183(7)
Ag(1)	3437(1)	8084(1)	2620(1)	41(1)
Ag(1')	4429(2)	8696(1)	3628(3)	52(1)
C(1)	2861(5)	7189(4)	1681(6)	43(2)
C(2)	2628(7)	7532(5)	983(6)	53(3)
C(3)	1908(6)	7842(5)	778(6)	52(3)
C(4)	1408(6)	7771(5)	1261(7)	60(3)
C(5)	1640(6)	7436(5)	1978(7)	57(3)
C(6)	2355(5)	7147(4)	2200(6)	42(2)
C(7)	3537(5)	6796(4)	1848(5)	38(2)
C(8)	2631(5)	6756(5)	2936(7)	47(2)
C(11)	4319(5)	6906(4)	2201(5)	36(2)
C(12)	4666(4)	7461(4)	2464(5)	33(2)
C(13)	4595(6)	7937(5)	1948(7)	49(2)
C(14)	5024(7)	8422(5)	2252(8)	61(3)
C(15)	5520(7)	8462(6)	3063(8)	64(3)
C(16)	5598(7)	7986(4)	3598(7)	47(2)
C(17)	5170(5)	7500(4)	3308(5)	34(2)
C(18)	5141(4)	6988(4)	3799(5)	33(2)
C(19)	5337(4)	6483(4)	3398(5)	34(2)
C(20)	5855(5)	6054(4)	3760(6)	41(2)
C(21)	5975(7)	5593(5)	3280(7)	53(3)
C(22)	5534(7)	5555(5)	2449(8)	62(3)
C(23)	4996(7)	5986(5)	2060(6)	52(3)
C(24)	4899(5)	6442(4)	2533(5)	37(2)
C(31)	3125(5)	6865(4)	3691(6)	40(2)
C(32)	3418(5)	7447(4)	3997(5)	39(2)
C(33)	2940(7)	7913(5)	3969(7)	53(3)
C(34)	3264(9)	8419(6)	4331(7)	78(4)
C(35)	4076(8)	8466(5)	4766(6)	59(3)
C(36)	4550(7)	7998(5)	4805(6)	50(2)
C(37)	4242(5)	7504(4)	4417(5)	32(2)
C(38)	4700(4)	6978(3)	4336(5)	29(2)
C(39)	4378(5)	6466(4)	4608(5)	36(2)
C(40)	4821(6)	6039(4)	5120(5)	41(2)
C(41)	4436(8)	5579(5)	5338(7)	59(3)
C(42)	3610(8)	5543(6)	5041(8)	64(3)
C(43)	3176(6)	5956(5)	4534(8)	58(3)
C(44)	3539(5)	6418(4)	4309(6)	44(2)

Table S15: Atomic coordinates (× 10^4) and equivalent isotropic displacement parameters ($Å^2$ × 10^3). U(eq) is defined as one third of the trace of the orthogonalized U_{ij} tensor.

	x	y	z	U(eq)
C(51)	1935(11)	9164(8)	2204(12)	97(5)
C(52)	2569(9)	9226(7)	1883(11)	84(4)
O(1)	3322(5)	9069(4)	2491(6)	67(2)
C(53)	3860(20)	9434(15)	2950(20)	146(13)
C(53')	3400(50)	9642(16)	2540(60)	170(40)
C(54)	3930(20)	10026(16)	3090(20)	187(13)
Ag(2)	9383(1)	8675(1)	3620(1)	46(1)
Ag(2')	8469(1)	8064(1)	2643(1)	44(1)
C(61)	7853(5)	7138(4)	1665(5)	43(2)
C(62)	7607(7)	7489(5)	964(7)	56(3)
C(63)	6904(7)	7795(5)	767(7)	57(3)
C(64)	6437(6)	7734(5)	1274(6)	53(3)
C(65)	6666(6)	7402(6)	1993(7)	61(3)
C(66)	7378(6)	7099(4)	2199(6)	47(2)
C(67)	8539(6)	6742(5)	1826(6)	47(2)
C(68)	7633(5)	6696(5)	2915(7)	47(2)
C(71)	9314(6)	6873(4)	2178(5)	38(2)
C(72)	9645(6)	7426(4)	2443(6)	42(2)
C(73)	9570(7)	7899(5)	1920(7)	51(2)
C(74)	10003(8)	8408(5)	2245(9)	63(3)
C(75)	10490(7)	8434(5)	3034(8)	58(3)
C(76)	10569(7)	7976(5)	3593(7)	49(2)
C(77)	10144(5)	7473(4)	3298(6)	36(2)
C(78)	10113(5)	6962(4)	3781(5)	33(2)
C(79)	10326(5)	6449(4)	3388(5)	32(2)
C(80)	10871(5)	6026(4)	3767(6)	37(2)
C(81)	10982(6)	5578(5)	3300(7)	52(3)
C(82)	10569(7)	5533(5)	2462(7)	56(3)
C(83)	10019(7)	5936(5)	2072(6)	56(3)
C(84)	9890(5)	6399(4)	2526(5)	35(2)
C(91)	8122(5)	6830(4)	3693(6)	42(2)
C(92)	8405(6)	7402(4)	4007(5)	42(2)
C(93)	7873(7)	7851(5)	3945(6)	58(3)
C(94)	8181(9)	8357(6)	4339(7)	69(4)
C(95)	8966(9)	8402(5)	4784(7)	68(4)
C(96)	9517(7)	7963(4)	4817(5)	48(2)
C(97)	9210(6)	7452(4)	4412(6)	42(2)
C(98)	9685(5)	6947(4)	4330(5)	33(2)
C(99)	9378(5)	6439(4)	4609(5)	36(2)
C(100)	9821(5)	6012(4)	5124(5)	41(2)
C(101)	9436(8)	5545(5)	5323(7)	58(3)
C(102)	8608(8)	5493(6)	5032(8)	68(4)
C(103)	8161(7)	5914(6)	4499(8)	61(3)
C(104)	8536(5)	6374(4)	4290(6)	40(2)
C(111)	8940(30)	10050(30)	3280(30)	290(30)
C(112)	8810(40)	9660(30)	2770(40)	340(40)
O(2)	8412(7)	9099(6)	2604(8)	103(4)
C(113)	7705(15)	9097(12)	1997(17)	139(8)
C(114)	7030(10)	9166(8)	2253(11)	93(5)

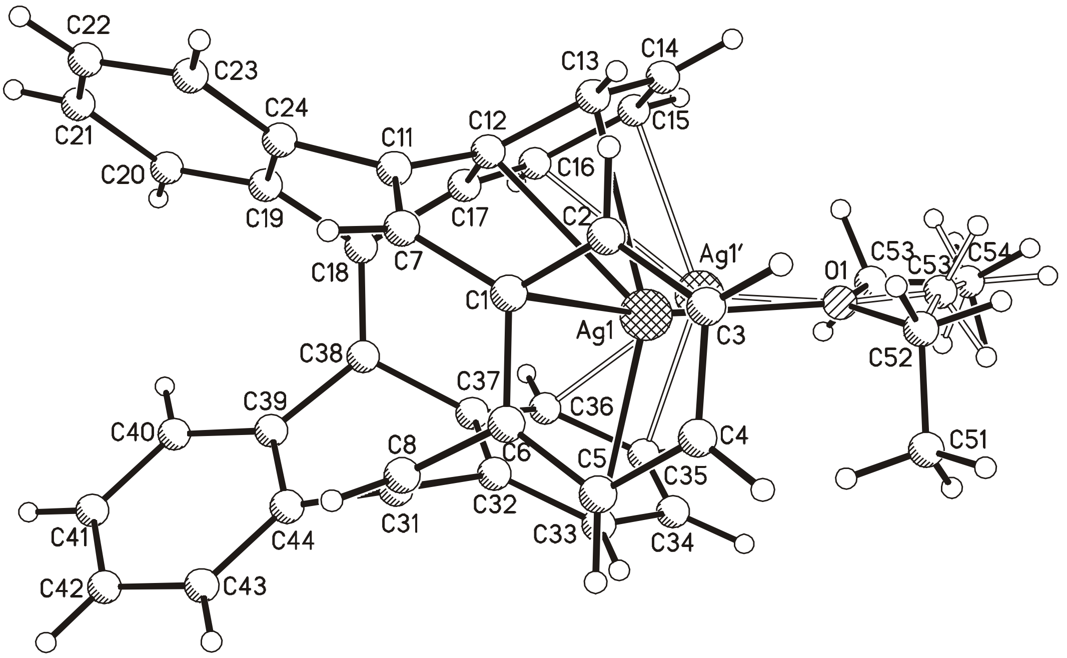

Table S16: Bond lengths [Å].

Ag(1)-O(1)	2.345(8)	Ag(1')-C(35)	2.251(13)
Ag(1)-C(1)	2.647(9)	Ag(1')-O(1)	2.434(10)
Ag(1)-C(13)	2.654(10)	Ag(1')-C(15)	2.468(14)
Ag(1)-C(33)	2.703(10)	Ag(1')-C(36)	2.531(11)
Ag(1)-C(12)	2.708(8)	Ag(1')-C(16)	2.681(12)
C(1)-C(2)	1.377(14)	C(22)-C(23)	1.411(15)
C(1)-C(6)	1.436(14)	C(23)-C(24)	1.382(13)
C(1)-C(7)	1.472(13)	C(31)-C(32)	1.505(14)
C(2)-C(3)	1.418(15)	C(31)-C(44)	1.505(13)
C(3)-C(4)	1.384(17)	C(32)-C(33)	1.384(14)
C(4)-C(5)	1.391(17)	C(32)-C(37)	1.414(11)
C(5)-C(6)	1.384(14)	C(33)-C(34)	1.38(2)
C(6)-C(8)	1.497(14)	C(34)-C(35)	1.401(19)
C(7)-C(11)	1.352(13)	C(35)-C(36)	1.380(15)
C(8)-C(31)	1.320(14)	C(36)-C(37)	1.367(14)
C(11)-C(12)	1.459(13)	C(37)-C(38)	1.514(12)
C(11)-C(24)	1.489(12)	C(38)-C(39)	1.473(12)
C(12)-C(13)	1.403(13)	C(39)-C(40)	1.397(13)
C(12)-C(17)	1.422(11)	C(39)-C(44)	1.416(12)
C(13)-C(14)	1.384(16)	C(40)-C(41)	1.395(14)
C(14)-C(15)	1.372(17)	C(41)-C(42)	1.394(16)
C(15)-C(16)	1.421(16)	C(42)-C(43)	1.366(17)
C(16)-C(17)	1.379(13)	C(43)-C(44)	1.379(14)
C(17)-C(18)	1.476(12)	C(51)-C(52)	1.40(2)
C(18)-C(38)	1.366(12)	C(52)-O(1)	1.452(17)
C(18)-C(19)	1.465(13)	C(52)-C(53')	1.83(5)
C(19)-C(20)	1.377(13)	O(1)-C(53)	1.34(3)
C(19)-C(24)	1.419(11)	O(1)-C(53')	1.36(4)
C(20)-C(21)	1.412(14)	C(53)-C(54)	1.42(4)
C(21)-C(22)	1.372(16)	C(53')-C(54)	1.42(4)

Table S17: Bond angles [°].

O(1)-Ag(1)-C(1)	137.3(3)	C(35)-Ag(1')-O(1)	112.5(4)
O(1)-Ag(1)-C(13)	98.5(3)	C(35)-Ag(1')-C(15)	136.8(5)
C(1)-Ag(1)-C(13)	81.3(3)	O(1)-Ag(1')-C(15)	108.4(4)
O(1)-Ag(1)-C(33)	100.6(4)	C(35)-Ag(1')-C(36)	32.9(4)
C(1)-Ag(1)-C(33)	102.3(4)	O(1)-Ag(1')-C(36)	134.4(4)
C(13)-Ag(1)-C(33)	146.5(3)	C(15)-Ag(1')-C(36)	105.5(4)
O(1)-Ag(1)-C(12)	125.6(3)	C(35)-Ag(1')-C(16)	106.6(4)
C(1)-Ag(1)-C(12)	71.3(3)	O(1)-Ag(1')-C(16)	130.6(4)
C(13)-Ag(1)-C(12)	30.3(3)	C(15)-Ag(1')-C(16)	31.7(4)
C(33)-Ag(1)-C(12)	118.8(3)	C(36)-Ag(1')-C(16)	74.2(3)
C(2)-C(1)-C(6)	118.5(9)	C(8)-C(31)-C(32)	124.2(9)
C(2)-C(1)-C(7)	123.1(10)	C(8)-C(31)-C(44)	123.9(10)
C(6)-C(1)-C(7)	117.7(8)	C(32)-C(31)-C(44)	111.5(8)
C(1)-C(2)-C(3)	121.4(11)	C(33)-C(32)-C(37)	117.8(9)
C(4)-C(3)-C(2)	119.1(10)	C(33)-C(32)-C(31)	125.2(9)
C(3)-C(4)-C(5)	120.3(10)	C(37)-C(32)-C(31)	116.7(8)
C(6)-C(5)-C(4)	120.8(11)	C(32)-C(33)-C(34)	120.7(11)
C(5)-C(6)-C(1)	119.7(9)	C(33)-C(34)-C(35)	121.0(11)
C(5)-C(6)-C(8)	124.3(10)	C(36)-C(35)-C(34)	118.2(11)
C(1)-C(6)-C(8)	116.0(8)	C(37)-C(36)-C(35)	121.2(10)
C(11)-C(7)-C(1)	128.6(9)	C(36)-C(37)-C(32)	121.0(9)
C(31)-C(8)-C(6)	128.2(10)	C(36)-C(37)-C(38)	126.8(8)
C(7)-C(11)-C(12)	125.7(8)	C(32)-C(37)-C(38)	112.1(7)
C(7)-C(11)-C(24)	121.0(9)	C(18)-C(38)-C(39)	125.1(8)
C(12)-C(11)-C(24)	112.0(7)	C(18)-C(38)-C(37)	118.6(7)
C(13)-C(12)-C(17)	117.9(9)	C(39)-C(38)-C(37)	112.3(7)
C(13)-C(12)-C(11)	126.1(8)	C(40)-C(39)-C(44)	119.0(8)
C(17)-C(12)-C(11)	115.8(8)	C(40)-C(39)-C(38)	126.0(8)
C(14)-C(13)-C(12)	120.5(10)	C(44)-C(39)-C(38)	114.9(8)
C(15)-C(14)-C(13)	122.0(11)	C(41)-C(40)-C(39)	120.0(9)
C(14)-C(15)-C(16)	118.5(11)	C(42)-C(41)-C(40)	119.9(10)
C(17)-C(16)-C(15)	120.3(10)	C(43)-C(42)-C(41)	120.2(10)
C(16)-C(17)-C(12)	120.7(9)	C(42)-C(43)-C(44)	121.2(10)
C(16)-C(17)-C(18)	126.9(8)	C(43)-C(44)-C(39)	119.7(9)
C(12)-C(17)-C(18)	112.3(7)	C(43)-C(44)-C(31)	125.3(9)
C(38)-C(18)-C(19)	123.8(8)	C(39)-C(44)-C(31)	114.8(8)
C(38)-C(18)-C(17)	120.6(8)	C(51)-C(52)-O(1)	112.1(15)
C(19)-C(18)-C(17)	111.1(7)	C(51)-C(52)-C(53')	115(4)
C(20)-C(19)-C(24)	118.4(8)	O(1)-C(52)-C(53')	47.5(16)
C(20)-C(19)-C(18)	128.3(8)	C(53)-O(1)-C(53')	44(3)
C(24)-C(19)-C(18)	113.2(7)	C(53)-O(1)-C(52)	125.0(17)
C(19)-C(20)-C(21)	121.2(9)	C(53')-O(1)-C(52)	81(3)
C(22)-C(21)-C(20)	119.3(9)	O(1)-C(53)-C(54)	138(3)
C(21)-C(22)-C(23)	120.8(10)	O(1)-C(53')-C(54)	135(4)
C(24)-C(23)-C(22)	119.1(9)	O(1)-C(53')-C(52)	51.7(17)
C(23)-C(24)-C(19)	121.0(8)	C(54)-C(53')-C(52)	169(8)
C(23)-C(24)-C(11)	123.8(8)	C(53)-C(54)-C(53')	42(3)
C(19)-C(24)-C(11)	115.1(8)		

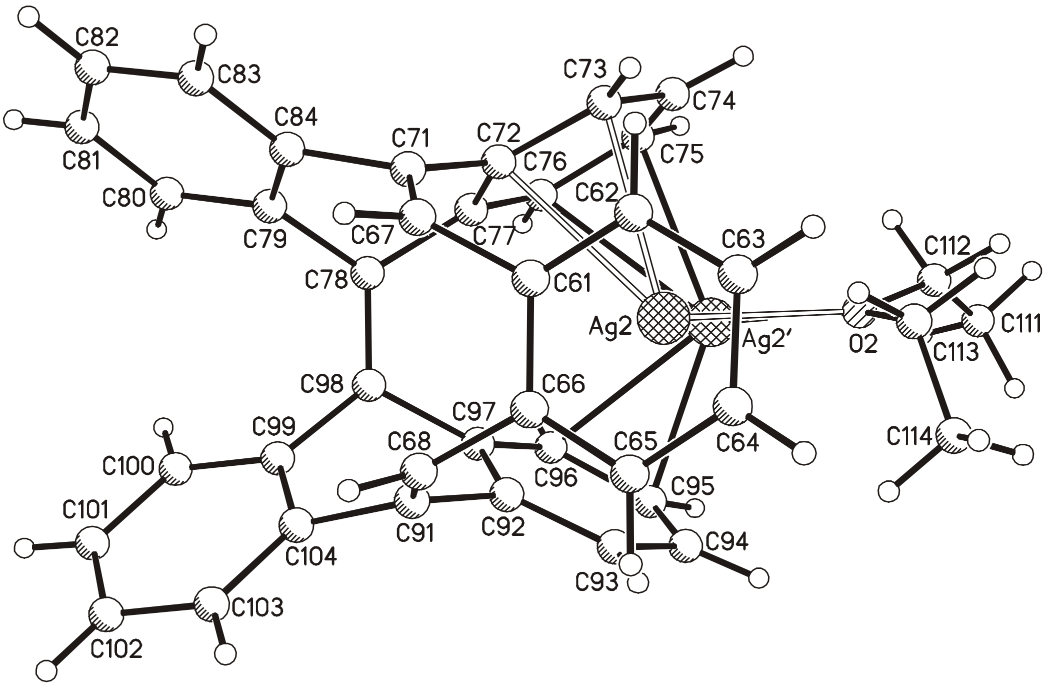

Table S18: Bond lengths [Å].

Ag(2)-O(2)	2.245(10)	Ag(2)-C(76)	2.687(11)
Ag(2)-C(95)	2.383(13)	Ag(2')-O(2)	2.452(14)
Ag(2)-C(75)	2.518(11)	Ag(2')-C(73)	2.628(10)
Ag(2)-C(96)	2.577(10)	Ag(2')-C(72)	2.679(9)
C(61)-C(62)	1.394(14)	C(81)-C(82)	1.371(15)
C(61)-C(66)	1.410(15)	C(82)-C(83)	1.374(15)
C(61)-C(67)	1.492(12)	C(83)-C(84)	1.395(13)
C(62)-C(63)	1.390(14)	C(91)-C(92)	1.484(15)
C(63)-C(64)	1.366(17)	C(91)-C(104)	1.497(13)
C(64)-C(65)	1.391(17)	C(92)-C(97)	1.380(14)
C(65)-C(66)	1.398(13)	C(92)-C(93)	1.403(13)
C(66)-C(68)	1.491(14)	C(93)-C(94)	1.39(2)
C(67)-C(71)	1.350(14)	C(94)-C(95)	1.360(19)
C(68)-C(91)	1.359(15)	C(95)-C(96)	1.416(16)
C(71)-C(72)	1.447(14)	C(96)-C(97)	1.409(14)
C(71)-C(84)	1.506(12)	C(97)-C(98)	1.493(12)
C(72)-C(73)	1.404(14)	C(98)-C(99)	1.455(13)
C(72)-C(77)	1.435(13)	C(99)-C(100)	1.399(12)
C(73)-C(74)	1.440(17)	C(99)-C(104)	1.428(12)
C(74)-C(75)	1.336(18)	C(100)-C(101)	1.394(15)
C(75)-C(76)	1.412(16)	C(101)-C(102)	1.400(16)
C(76)-C(77)	1.412(14)	C(102)-C(103)	1.406(17)
C(77)-C(78)	1.468(12)	C(103)-C(104)	1.379(15)
C(78)-C(98)	1.366(12)	C(111)-C(112)	1.23(7)
C(78)-C(79)	1.485(12)	C(112)-O(2)	1.48(7)
C(79)-C(80)	1.399(11)	O(2)-C(113)	1.35(3)
C(79)-C(84)	1.416(11)	C(113)-C(114)	1.40(3)
C(80)-C(81)	1.368(15)		

Table S19: Bond angles [°].

O(2)-Ag(2)-C(95)	112.5(5)	C(77)-C(78)-C(79)	111.9(7)
O(2)-Ag(2)-C(75)	108.1(5)	C(80)-C(79)-C(84)	119.0(8)
C(95)-Ag(2)-C(75)	137.5(4)	C(80)-C(79)-C(78)	128.3(8)
O(2)-Ag(2)-C(96)	138.3(4)	C(84)-C(79)-C(78)	112.8(7)
C(95)-Ag(2)-C(96)	32.9(4)	C(81)-C(80)-C(79)	119.8(8)
C(75)-Ag(2)-C(96)	105.2(4)	C(80)-C(81)-C(82)	121.4(9)
O(2)-Ag(2)-C(76)	132.9(4)	C(81)-C(82)-C(83)	120.4(10)
C(95)-Ag(2)-C(76)	106.9(4)	C(82)-C(83)-C(84)	119.9(9)
C(75)-Ag(2)-C(76)	31.3(3)	C(83)-C(84)-C(79)	119.5(8)
C(96)-Ag(2)-C(76)	74.2(3)	C(83)-C(84)-C(71)	125.5(8)
O(2)-Ag(2')-C(73)	99.6(4)	C(79)-C(84)-C(71)	115.0(7)
O(2)-Ag(2')-C(72)	126.0(4)	C(68)-C(91)-C(92)	126.7(9)
C(73)-Ag(2')-C(72)	30.7(3)	C(68)-C(91)-C(104)	120.4(10)
C(62)-C(61)-C(66)	119.0(8)	C(92)-C(91)-C(104)	112.3(8)
C(62)-C(61)-C(67)	123.4(9)	C(97)-C(92)-C(93)	122.6(11)
C(66)-C(61)-C(67)	117.2(9)	C(97)-C(92)-C(91)	115.9(8)
C(63)-C(62)-C(61)	122.1(11)	C(93)-C(92)-C(91)	121.4(10)
C(64)-C(63)-C(62)	118.0(10)	C(94)-C(93)-C(92)	117.5(11)
C(63)-C(64)-C(65)	122.0(9)	C(95)-C(94)-C(93)	120.6(11)
C(64)-C(65)-C(66)	120.0(11)	C(94)-C(95)-C(96)	122.5(11)
C(65)-C(66)-C(61)	118.8(10)	C(97)-C(96)-C(95)	117.0(10)
C(65)-C(66)-C(68)	123.4(10)	C(92)-C(97)-C(96)	119.7(9)
C(61)-C(66)-C(68)	117.6(8)	C(92)-C(97)-C(98)	114.2(9)
C(71)-C(67)-C(61)	126.5(10)	C(96)-C(97)-C(98)	126.0(9)
C(91)-C(68)-C(66)	124.5(10)	C(78)-C(98)-C(99)	125.4(8)
C(67)-C(71)-C(72)	127.0(8)	C(78)-C(98)-C(97)	119.4(8)
C(67)-C(71)-C(84)	118.2(9)	C(99)-C(98)-C(97)	110.8(8)
C(72)-C(71)-C(84)	113.1(8)	C(100)-C(99)-C(104)	118.2(9)
C(73)-C(72)-C(77)	118.1(10)	C(100)-C(99)-C(98)	127.0(8)
C(73)-C(72)-C(71)	125.5(9)	C(104)-C(99)-C(98)	114.8(7)
C(77)-C(72)-C(71)	116.3(8)	C(101)-C(100)-C(99)	119.9(9)
C(72)-C(73)-C(74)	119.7(10)	C(100)-C(101)-C(102)	121.7(9)
C(75)-C(74)-C(73)	121.0(10)	C(101)-C(102)-C(103)	118.7(10)
C(74)-C(75)-C(76)	121.5(11)	C(104)-C(103)-C(102)	120.1(10)
C(77)-C(76)-C(75)	118.8(11)	C(103)-C(104)-C(99)	121.3(9)
C(76)-C(77)-C(72)	120.7(9)	C(103)-C(104)-C(91)	124.4(8)
C(76)-C(77)-C(78)	127.6(9)	C(99)-C(104)-C(91)	114.2(8)
C(72)-C(77)-C(78)	111.7(8)	C(111)-C(112)-O(2)	141(8)
C(98)-C(78)-C(77)	121.1(8)	C(113)-O(2)-C(112)	115(3)
C(98)-C(78)-C(79)	123.4(8)	O(2)-C(113)-C(114)	117(2)

Table S20: Bond lengths [Å] and angles [°].

Sb(1)-F(6)	1.694(11)	Sb(2)-F(16)	1.782(12)
Sb(1)-F(3)	1.824(9)	Sb(2)-F(14)	1.817(10)
Sb(1)-F(5)	1.855(8)	Sb(2)-F(11)	1.833(8)
Sb(1)-F(1)	1.876(7)	Sb(2)-F(12)	1.864(9)
Sb(1)-F(2)	1.895(7)	Sb(2)-F(15)	1.875(10)
Sb(1)-F(4)	1.924(9)	Sb(2)-F(13)	1.960(10)
F(6)-Sb(1)-F(3)	99.6(8)	F(16)-Sb(2)-F(14)	93.3(6)
F(6)-Sb(1)-F(5)	88.1(8)	F(16)-Sb(2)-F(11)	91.4(5)
F(3)-Sb(1)-F(5)	172.3(7)	F(14)-Sb(2)-F(11)	89.3(4)
F(6)-Sb(1)-F(1)	90.0(6)	F(16)-Sb(2)-F(12)	95.1(5)
F(3)-Sb(1)-F(1)	90.1(4)	F(14)-Sb(2)-F(12)	93.0(4)
F(5)-Sb(1)-F(1)	89.6(4)	F(11)-Sb(2)-F(12)	172.9(6)
F(6)-Sb(1)-F(2)	90.6(6)	F(16)-Sb(2)-F(15)	105.9(7)
F(3)-Sb(1)-F(2)	89.2(4)	F(14)-Sb(2)-F(15)	160.4(7)
F(5)-Sb(1)-F(2)	91.0(4)	F(11)-Sb(2)-F(15)	86.3(4)
F(1)-Sb(1)-F(2)	179.2(5)	F(12)-Sb(2)-F(15)	89.3(4)
F(6)-Sb(1)-F(4)	172.9(8)	F(16)-Sb(2)-F(13)	173.3(6)
F(3)-Sb(1)-F(4)	87.5(6)	F(14)-Sb(2)-F(13)	80.5(5)
F(5)-Sb(1)-F(4)	84.8(6)	F(11)-Sb(2)-F(13)	85.8(4)
F(1)-Sb(1)-F(4)	89.4(4)	F(12)-Sb(2)-F(13)	88.0(4)
F(2)-Sb(1)-F(4)	90.0(4)	F(15)-Sb(2)-F(13)	80.1(5)

Table S21: Anisotropic displacement parameters ($Å^2 \times 10^3$). The anisotropic displacement factor exponent takes the form: $-2\pi^2[h^2 a^{*2}U_{11} + ... + 2 h k a^* b^* U_{12}]$.

	U_{11}	U_{22}	U_{33}	U_{23}	U_{13}	U_{12}
Sb(1)	73(1)	40(1)	54(1)	-3(1)	25(1)	-2(1)
F(1)	120(6)	33(3)	95(6)	-2(4)	22(5)	-4(4)
F(2)	149(8)	38(4)	84(5)	-11(4)	32(5)	-4(5)
F(3)	213(13)	64(6)	49(4)	6(3)	21(6)	31(6)
F(4)	72(5)	66(5)	126(8)	22(5)	2(5)	-7(4)
F(5)	203(12)	95(7)	62(5)	1(4)	70(7)	-2(7)
F(6)	78(7)	144(12)	267(19)	12(10)	81(9)	14(6)
Sb(2)	134(2)	41(1)	69(1)	-5(1)	56(1)	-7(1)
F(11)	174(11)	48(5)	162(12)	-26(6)	28(9)	-20(6)
F(12)	214(15)	58(5)	108(8)	-16(5)	19(9)	16(7)
F(13)	135(10)	94(8)	175(12)	12(7)	55(9)	-27(7)
F(14)	195(15)	108(10)	136(11)	25(7)	-33(10)	9(9)
F(15)	264(19)	102(10)	157(12)	35(8)	134(13)	60(10)
Ag(1)	44(1)	37(1)	39(1)	-5(1)	9(1)	1(1)
Ag(1')	51(2)	53(2)	52(2)	-3(1)	17(1)	-3(1)
C(1)	39(5)	43(5)	34(4)	-7(4)	-7(4)	6(4)
C(2)	69(7)	53(6)	36(5)	-4(4)	14(5)	5(5)
C(3)	45(5)	65(7)	33(4)	-2(4)	-6(4)	12(5)
C(4)	49(6)	58(7)	55(6)	-6(5)	-8(5)	19(5)
C(5)	40(5)	74(8)	54(6)	0(5)	12(5)	4(5)
C(6)	32(4)	54(6)	32(4)	-6(4)	-2(4)	1(4)
C(7)	35(4)	48(5)	29(4)	-1(4)	6(4)	-1(4)
C(8)	25(4)	64(7)	49(6)	3(5)	5(4)	-8(4)
C(11)	33(4)	46(5)	28(4)	0(3)	10(4)	6(3)
C(12)	24(4)	47(5)	28(4)	4(3)	10(3)	0(3)
C(13)	47(5)	57(6)	49(6)	13(5)	22(5)	0(5)
C(14)	70(7)	52(7)	63(7)	14(5)	23(6)	-10(5)
C(15)	61(7)	59(7)	69(8)	4(6)	14(6)	-20(6)
C(16)	55(6)	45(6)	46(5)	-3(4)	22(5)	-7(5)
C(17)	24(4)	42(5)	36(4)	4(4)	11(3)	-8(3)
C(18)	18(3)	48(5)	29(4)	9(3)	1(3)	-4(3)
C(19)	21(4)	48(5)	33(4)	-2(4)	7(3)	1(3)
C(20)	35(5)	49(5)	35(4)	5(4)	7(4)	-1(4)
C(21)	57(6)	50(6)	54(6)	2(5)	20(5)	19(5)
C(22)	67(7)	55(7)	61(7)	-16(5)	17(6)	26(6)
C(23)	65(6)	59(6)	31(4)	-2(4)	16(4)	24(5)
C(24)	30(4)	49(5)	27(4)	2(4)	2(3)	5(4)
C(31)	26(4)	58(6)	38(5)	6(4)	14(4)	2(4)
C(32)	32(4)	56(6)	28(4)	0(4)	9(3)	7(4)
C(33)	53(6)	64(7)	49(6)	3(5)	26(5)	14(5)
C(34)	106(11)	79(9)	45(6)	-4(6)	19(7)	46(8)
C(35)	88(8)	52(6)	31(4)	-11(4)	10(5)	14(6)
C(36)	63(6)	57(6)	30(4)	-6(4)	13(5)	0(5)
C(37)	28(4)	47(5)	17(3)	3(3)	2(3)	8(3)
C(38)	21(3)	40(4)	20(3)	-1(3)	-1(3)	-4(3)
C(39)	39(4)	44(5)	26(4)	0(3)	14(4)	-6(4)
C(40)	40(5)	50(5)	31(4)	8(4)	7(4)	0(4)
C(41)	82(8)	49(6)	47(6)	21(5)	21(6)	-8(5)
C(42)	57(7)	68(8)	66(8)	16(6)	17(6)	-14(6)
C(43)	44(5)	66(7)	70(7)	9(5)	26(5)	-15(5)
C(44)	36(4)	55(6)	43(5)	12(4)	14(4)	-11(4)

Table S22: Anisotropic displacement parameters ($Å^2 \times 10^3$). The anisotropic displacement factor exponent takes the form: $-2\pi^2[h^2 a^{*2}U_{11} + ... + 2 h k a^* b^* U_{12}]$.

	U_{11}	U_{22}	U_{33}	U_{23}	U_{13}	U_{12}
C(51)	96(12)	95(12)	105(13)	9(10)	40(10)	-11(10)
C(52)	67(8)	83(10)	97(11)	-12(8)	16(8)	-8(7)
O(1)	59(5)	54(5)	91(6)	15(4)	30(5)	6(4)
Ag(2)	53(1)	40(1)	45(1)	2(1)	14(1)	2(1)
Ag(2')	44(1)	43(1)	42(1)	-3(1)	9(1)	5(1)
C(61)	31(4)	58(6)	30(4)	-8(4)	-6(4)	13(4)
C(62)	55(6)	68(7)	39(5)	6(5)	8(5)	32(5)
C(63)	58(6)	54(6)	43(5)	0(4)	-7(5)	26(5)
C(64)	34(5)	64(7)	47(5)	-3(5)	-9(4)	22(4)
C(65)	34(5)	91(9)	46(6)	-6(6)	-5(4)	21(5)
C(66)	39(5)	48(6)	45(5)	3(4)	1(4)	14(4)
C(67)	55(6)	47(5)	31(4)	-6(4)	2(4)	23(5)
C(68)	23(4)	58(6)	57(6)	6(5)	10(4)	3(4)
C(71)	39(5)	50(6)	26(4)	5(3)	12(4)	10(4)
C(72)	47(5)	45(5)	42(5)	7(4)	22(4)	17(4)
C(73)	64(6)	51(6)	48(6)	16(5)	31(5)	15(5)
C(74)	84(8)	41(6)	84(9)	22(6)	54(7)	7(5)
C(75)	62(7)	45(6)	79(8)	6(5)	38(6)	-5(5)
C(76)	54(6)	45(6)	53(6)	2(5)	23(5)	-2(5)
C(77)	30(4)	38(5)	44(5)	-1(4)	16(4)	2(3)
C(78)	30(4)	36(4)	35(4)	6(3)	12(3)	7(3)
C(79)	26(4)	32(4)	34(4)	3(3)	6(3)	7(3)
C(80)	23(4)	48(5)	36(4)	7(4)	2(3)	14(4)
C(81)	43(5)	57(6)	54(6)	13(5)	14(5)	22(5)
C(82)	68(7)	55(7)	45(5)	-8(4)	17(5)	26(5)
C(83)	57(6)	71(7)	38(5)	-11(5)	11(5)	24(5)
C(84)	34(4)	40(5)	30(4)	-2(3)	5(3)	12(3)
C(91)	35(5)	50(6)	41(5)	11(4)	15(4)	5(4)
C(92)	46(5)	60(6)	29(4)	14(4)	22(4)	21(4)
C(93)	61(6)	71(8)	42(5)	9(5)	17(5)	40(6)
C(94)	97(10)	70(8)	49(6)	13(6)	36(7)	41(7)
C(95)	110(10)	59(7)	33(5)	-11(5)	19(6)	20(7)
C(96)	64(6)	51(6)	23(4)	-2(4)	5(4)	6(5)
C(97)	53(5)	45(5)	29(4)	7(4)	15(4)	17(4)
C(98)	21(4)	49(5)	26(4)	6(3)	3(3)	9(3)
C(99)	36(4)	43(5)	27(4)	6(3)	8(3)	4(4)
C(100)	38(5)	48(5)	32(4)	11(4)	2(4)	4(4)
C(101)	71(7)	51(6)	53(6)	28(5)	19(6)	19(5)
C(102)	66(7)	65(8)	74(8)	29(6)	22(7)	-11(6)
C(103)	45(5)	74(8)	66(7)	26(6)	17(5)	-5(5)
C(104)	23(4)	58(6)	39(5)	4(4)	7(3)	1(4)
O(2)	68(6)	136(10)	100(8)	52(8)	19(5)	48(7)

Table S23: Hydrogen coordinates ($\times 10^4$) and isotropic displacement parameters ($Å^2 \times 10^3$).

	x	y	z	U(eq)
H(2)	2956	7561	633	64
H(3)	1771	8095	315	62
H(4)	904	7951	1102	71
H(5)	1303	7405	2319	68
H(7)	3405	6415	1688	46
H(8)	2424	6383	2849	57
H(13)	4251	7926	1386	59
H(14)	4973	8738	1889	73
H(15)	5805	8801	3262	77
H(16)	5946	8002	4157	57
H(20)	6136	6068	4343	49
H(21)	6358	5311	3531	64
H(22)	5592	5234	2132	74
H(23)	4704	5964	1480	62
H(33)	2384	7886	3698	63
H(34)	2930	8740	4284	94
H(35)	4296	8811	5026	71
H(36)	5101	8019	5107	60
H(40)	5384	6064	5320	50
H(41)	4737	5290	5689	71
H(42)	3348	5229	5192	77
H(43)	2613	5925	4332	70
H(51A)	1439	9271	1774	145
H(51B)	2019	9407	2696	145
H(51C)	1901	8769	2366	145
H(52A)	2597	9624	1714	101
H(52B)	2474	8987	1377	101
H(53A)	4342	9343	2793	175
H(53B)	3974	9284	3529	175
H(53C)	2871	9771	2550	200
H(53D)	3425	9749	1978	200
H(54A)	4493	10127	3337	281
H(54B)	3635	10139	3465	281
H(54C)	3715	10222	2548	281
H(54D)	4474	9953	3078	281
H(54E)	3909	9977	3658	281
H(54F)	3781	10414	2900	281
H(62)	7931	7520	609	67
H(63)	6751	8039	294	68
H(64)	5941	7925	1131	64
H(65)	6338	7381	2345	73
H(67)	8419	6359	1666	56
H(68)	7440	6319	2822	56
H(73)	9235	7884	1355	62
H(74)	9942	8730	1893	76
H(75)	10789	8768	3223	70
H(76)	10902	8005	4158	59
H(80)	11163	6049	4347	45
H(81)	11353	5292	3561	62
H(82)	10664	5222	2149	68
H(83)	9726	5898	1494	68

Table S24: Hydrogen coordinates (× 10^4) and isotropic displacement parameters (Å^2 × 10^3).

	x	y	z	U(eq)
H(93)	7324	7812	3646	70
H(94)	7841	8673	4296	83
H(95)	9150	8742	5083	82
H(96)	10068	8010	5100	58
H(100)	10383	6041	5337	49
H(101)	9743	5255	5665	70
H(102)	8353	5179	5191	81
H(103)	7599	5881	4284	74
H(11A)	9132	10377	3041	437
H(11B)	9352	9931	3795	437
H(11C)	8455	10143	3401	437
H(11D)	9354	9563	2757	409
H(11E)	8562	9857	2232	409
H(11F)	7653	8735	1690	166
H(11G)	7709	9402	1595	166
H(11H)	6554	9070	1789	140
H(11I)	6996	9560	2420	140
H(11J)	7068	8918	2731	140

3.4 Compound 5b

3.4.1 Sideview

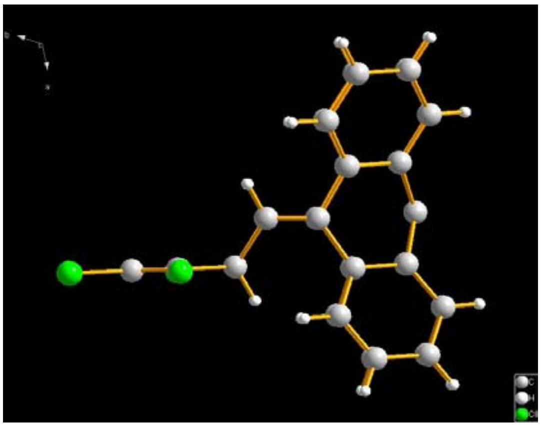

3.4.2 Frontview

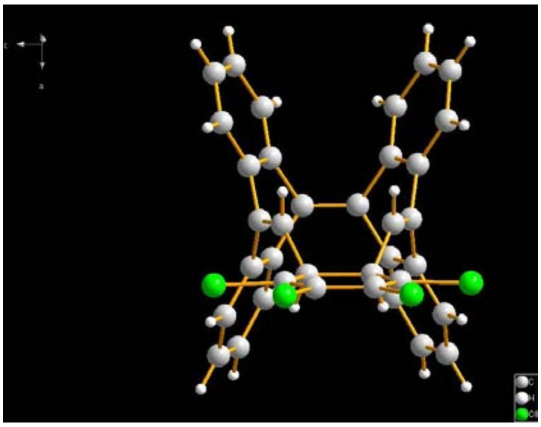

3.4.3 Topview

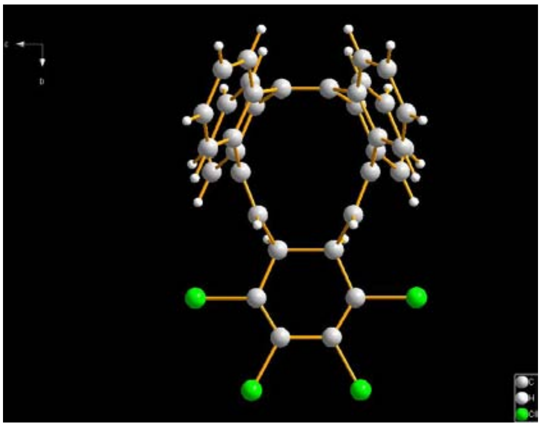

3.4.4 Data sheets

Table S25: Crystal data and structure refinement for compound **5b** (Benzene solvate, herges48).

Identification code	herges48	
Empirical formula	$C_{42}H_{25}Cl_4$	
Formula weight	671.42	
Temperature	170(2) K	
Wavelength	0.71073 Å	
Crystal system	hexagonal	
Space group	$P6_3/m$	
Unit cell dimensions	a = 19.5210(10) Å	α= 90°.
	b = 19.5210(10) Å	β= 90°.
	c = 15.7070(7) Å	γ = 120°.
Volume	5183.6(4) $Å^3$	
Z	6	
Density (calculated)	1.291 Mg/m^3	
Absorption coefficient	0.372 mm^{-1}	
F(000)	2070	
Crystal size	0.4 x 0.1 x 0.1 mm^3	
Theta range for data collection	2.41 to 25.02°.	
Index ranges	-23<=h<=23, -23<=k<=19, -18<=l<=18	
Reflections collected	28215	
Independent reflections	3169 [R(int) = 0.0550]	
Completeness to theta = 25.02°	99.7 %	
Refinement method	Full-matrix least-squares on F^2	
Data / restraints / parameters	3169 / 0 / 212	
Goodness-of-fit on F^2	1.062	
Final R indices [I>2sigma(I)]	R1 = 0.0572, wR2 = 0.1410	
R indices (all data)	R1 = 0.0722, wR2 = 0.1503	
Extinction coefficient	0.0049(10)	
Largest diff. peak and hole	0.498 and -0.344 e.$Å^{-3}$	

Comments:

All non-hydrogen atoms were refined anisotropically. All H atoms were positioned with idealized geometry and refined isotropically using a riding model. The molecule is located on a crystallographic mirror plane. The structure contains one benzene molecule which is also located on a crystallographic mirror plane. There is one additional benzene molecule, which is

completely disordered and therefore, the data were corrected for disordered solvent using the SQUEEZE option in Platon.

Table S26: Atomic coordinates (× 10^4) and equivalent isotropic displacement parameters (Å^2 × 10^3). U(eq) is defined as one third of the trace of the orthogonalized U_{ij} tensor.

	x	y	z	U(eq)
C(1)	3202(2)	485(2)	6327(2)	31(1)
C(2)	2848(2)	-388(2)	6232(2)	31(1)
C(3)	3196(2)	-758(2)	5796(2)	36(1)
C(4)	2811(2)	-1565(2)	5724(2)	42(1)
C(5)	2051(2)	-2036(2)	6070(2)	43(1)
C(6)	1689(2)	-1676(2)	6495(2)	36(1)
C(7)	2083(2)	-860(2)	6584(2)	30(1)
C(8)	1802(1)	-391(2)	7069(2)	27(1)
C(9)	1878(2)	295(2)	6563(2)	29(1)
C(10)	1315(2)	522(2)	6467(2)	34(1)
C(11)	1482(2)	1206(2)	6014(2)	41(1)
C(12)	2227(2)	1669(2)	5666(2)	41(1)
C(13)	2792(2)	1453(2)	5749(2)	36(1)
C(14)	2630(2)	761(2)	6188(2)	30(1)
C(15)	3926(2)	979(2)	6637(2)	33(1)
C(16)	4507(2)	758(2)	6994(2)	34(1)
C(17)	5314(2)	1276(2)	6603(2)	38(1)
Cl(1)	6865(1)	2281(1)	6514(1)	58(1)
C(18)	5978(2)	1711(2)	7035(2)	36(1)
Cl(2)	5314(1)	1248(1)	5500(1)	67(1)
C(21)	-370(5)	-3598(4)	7500	103(3)
C(22)	339(4)	-4036(3)	6730(4)	116(3)
C(23)	-144(4)	-3746(3)	6788(4)	100(2)
C(24)	645(6)	-4205(4)	7500	150(6)

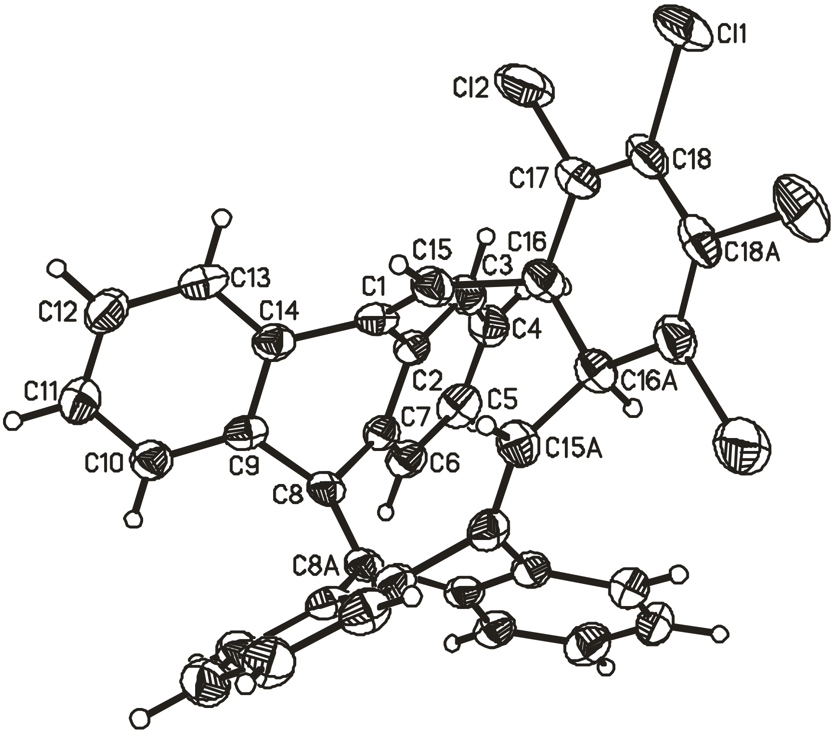

Table S27: Bond lengths [Å] and angles [°].

C(1)-C(15)	1.342(4)	C(11)-C(12)	1.384(5)
C(1)-C(14)	1.479(4)	C(12)-C(13)	1.371(4)
C(1)-C(2)	1.492(4)	C(13)-C(14)	1.405(4)
C(2)-C(3)	1.394(4)	C(15)-C(16)	1.509(4)
C(2)-C(7)	1.417(4)	C(16)-C(17)	1.513(4)
C(3)-C(4)	1.369(4)	C(16)-C(16A)	1.590(6)
C(4)-C(5)	1.406(5)	C(17)-C(18)	1.328(4)
C(5)-C(6)	1.393(4)	C(17)-Cl(2)	1.733(3)
C(6)-C(7)	1.386(4)	Cl(1)-C(18)	1.725(3)
C(7)-C(8)	1.492(4)	C(18)-C(18A)	1.462(6)
C(8)-C(8A)	1.355(5)	C(21)-C(23A)	1.288(8)
C(8)-C(9)	1.499(4)	C(21)-C(23)	1.288(8)
C(9)-C(10)	1.382(4)	C(22)-C(23)	1.322(10)
C(9)-C(14)	1.412(4)	C(22)-C(24)	1.458(10)
C(10)-C(11)	1.401(4)	C(24)-C(22A)	1.458(10)
C(15)-C(1)-C(14)	120.5(2)	C(12)-C(11)-C(10)	119.3(3)
C(15)-C(1)-C(2)	125.2(3)	C(13)-C(12)-C(11)	120.5(3)
C(14)-C(1)-C(2)	113.5(2)	C(12)-C(13)-C(14)	120.9(3)
C(3)-C(2)-C(7)	118.8(3)	C(13)-C(14)-C(9)	119.1(3)
C(3)-C(2)-C(1)	125.0(2)	C(13)-C(14)-C(1)	125.0(2)
C(7)-C(2)-C(1)	116.1(2)	C(9)-C(14)-C(1)	115.8(2)
C(4)-C(3)-C(2)	120.5(3)	C(1)-C(15)-C(16)	126.9(3)
C(3)-C(4)-C(5)	120.9(3)	C(15)-C(16)-C(17)	109.7(2)
C(6)-C(5)-C(4)	119.4(3)	C(15)-C(16)-C(16A)	111.81(15)
C(7)-C(6)-C(5)	119.9(3)	C(17)-C(16)-C(16A)	113.96(16)
C(6)-C(7)-C(2)	120.5(3)	C(18)-C(17)-C(16)	125.3(3)
C(6)-C(7)-C(8)	126.4(2)	C(18)-C(17)-Cl(2)	121.0(2)
C(2)-C(7)-C(8)	113.0(2)	C(16)-C(17)-Cl(2)	113.7(2)
C(8A)-C(8)-C(7)	120.71(15)	C(17)-C(18)-C(18A)	120.73(18)
C(8A)-C(8)-C(9)	121.97(14)	C(17)-C(18)-Cl(1)	121.0(3)
C(7)-C(8)-C(9)	112.1(2)	C(18A)-C(18)-Cl(1)	118.30(11)
C(10)-C(9)-C(14)	119.0(3)	C(23A)-C(21)-C(23)	120.6(10)
C(10)-C(9)-C(8)	127.2(2)	C(23)-C(22)-C(24)	120.0(6)

C(14)-C(9)-C(8)	113.8(2)	C(21)-C(23)-C(22)	123.6(8)
C(9)-C(10)-C(11)	121.2(3)	C(22A)-C(24)-C(22)	112.1(9)

Symmetry transformations used to generate equivalent atoms: A = x,y,-z+3/2

Table S28: Anisotropic displacement parameters ($Å^2 \times 10^3$). The anisotropic displacement factor exponent takes the form: $-2\pi^2[h^2 a^{*2}U_{11} + ... + 2 h k a^* b^* U_{12}]$.

	U_{11}	U_{22}	U_{33}	U_{23}	U_{13}	U_{12}
C(1)	29(1)	34(1)	24(1)	3(1)	6(1)	12(1)
C(2)	31(1)	43(2)	21(1)	2(1)	1(1)	21(1)
C(3)	35(2)	45(2)	32(2)	-4(1)	0(1)	22(1)
C(4)	50(2)	57(2)	32(2)	-6(1)	1(1)	37(2)
C(5)	55(2)	38(2)	37(2)	-5(1)	-3(1)	24(1)
C(6)	39(2)	42(2)	26(1)	-1(1)	-1(1)	20(1)
C(7)	28(1)	39(2)	20(1)	-1(1)	-4(1)	16(1)
C(8)	16(1)	31(1)	27(1)	-1(1)	-2(1)	7(1)
C(9)	27(1)	29(1)	20(1)	-3(1)	-2(1)	6(1)
C(10)	31(1)	41(2)	30(2)	1(1)	0(1)	16(1)
C(11)	45(2)	48(2)	36(2)	5(1)	-2(1)	29(2)
C(12)	52(2)	44(2)	33(2)	8(1)	2(1)	28(2)
C(13)	38(2)	35(2)	25(1)	5(1)	6(1)	12(1)
C(14)	32(1)	35(1)	22(1)	-2(1)	0(1)	14(1)
C(15)	27(1)	31(1)	38(2)	3(1)	3(1)	12(1)
C(16)	25(1)	38(2)	39(2)	-1(1)	1(1)	14(1)
C(17)	26(1)	46(2)	41(2)	3(1)	6(1)	18(1)
Cl(1)	23(1)	60(1)	77(1)	15(1)	14(1)	11(1)
C(18)	19(1)	32(1)	55(2)	6(1)	5(1)	12(1)
Cl(2)	37(1)	112(1)	41(1)	5(1)	10(1)	29(1)
C(21)	90(6)	57(4)	130(9)	0	0	14(4)
C(22)	129(5)	54(3)	111(5)	-14(3)	62(4)	5(3)
C(23)	122(5)	54(3)	82(4)	4(3)	-7(4)	13(3)
C(24)	86(6)	35(4)	310(20)	0	0	14(4)

Table S29: Hydrogen coordinates ($\times 10^4$) and isotropic displacement parameters ($Å^2 \times 10^3$).

	x	y	z	U(eq)
H(3)	3704	-448	5547	44
H(4)	3061	-1809	5436	50
H(5)	1788	-2596	6014	51
H(6)	1172	-1988	6723	43
H(10)	806	208	6713	41
H(11)	1087	1351	5946	49
H(12)	2347	2139	5369	49
H(13)	3301	1776	5506	43
H(15)	4090	1526	6631	40
H(16)	4326	203	6808	41
H(21)	-707	-3377	7500	124
H(22)	486	-4134	6187	140
H(23)	-334	-3642	6276	120

3.5 Compound 6

3.5.1 Sideview

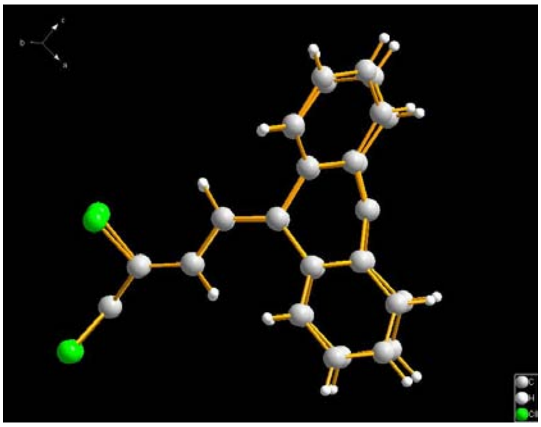

3.5.2 Frontview

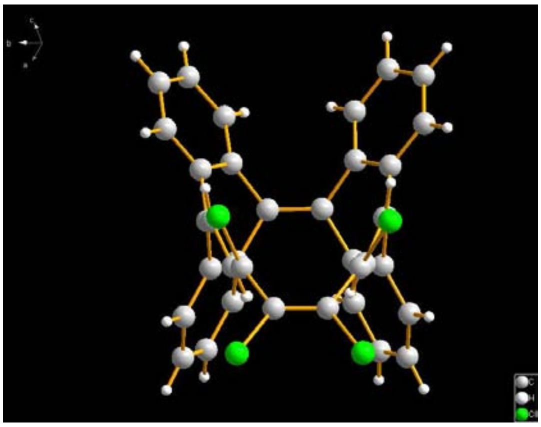

3.5.3 Topview

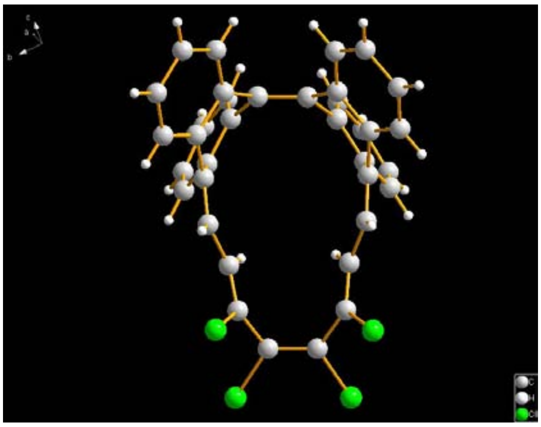

3.5.4 Data sheets

Table S30: Crystal data and structure refinement for $C_{36}H_{20}Cl_4$-chloroform solvate **6**.

Identification code	herges49	
Empirical formula	$C_{37}H_{21}Cl_7$	
Formula weight	713.69	
Temperature	293(2) K	
Wavelength	0.71073 Å	
Crystal system	monoclinic	
Space group	$P2_1/n$	
Unit cell dimensions	a = 13.2695(9) Å	α = 90°.
	b = 8.7070(3) Å	β = 90.036(8)°.
	c = 27.6283(18) Å	γ = 90°.
Volume	3192.1(3) Å^3	
Z	4	
Density (calculated)	1.485 Mg/m^3	
Absorption coefficient	0.650 mm^{-1}	
F(000)	1448	
Crystal size	0.13 x 0.10 x 0.07 mm^3	
Theta range for data collection	2.45 to 25.95°.	
Index ranges	-16<=h<=16, -9<=k<=10, -33<=l<=33	
Reflections collected	21311	
Independent reflections	6221 [R(int) = 0.0326]	
Completeness to theta = 25.95°	99.5 %	
Refinement method	Full-matrix least-squares on F^2	
Data / restraints / parameters	6221 / 0 / 414	
Goodness-of-fit on F^2	1.037	
Final R indices [I>2sigma(I)]	R1 = 0.0387, wR2 = 0.1003	
R indices (all data)	R1 = 0.0491, wR2 = 0.1057	
Extinction coefficient	0.0063(7)	
Largest diff. peak and hole	0.441 and -0.590 e.Å^{-3}	

Comments:

All non-hydrogen atoms were refined anisotropically. All H atoms were located by a difference map. The aromatic H atoms were positioned with idealized geometry and refined isotropically using a riding model. The H atoms at C2, C3, C8 and C9 were refined with varying coordinates and varying isotropic displacement parameters.

A numerical absorption correction was performed (Tmin/tmax.: 0.8192 / 0.9479).

Table 31: Atomic coordinates (× 10^4) and equivalent isotropic displacement parameters ($Å^2$ × 10^3). U(eq) is defined as one third of the trace of the orthogonalized U_{ij} tensor.

	x	y	z	U(eq)
C(1)	4027(1)	8883(2)	6606(1)	17(1)
C(2)	3374(2)	8112(2)	6320(1)	18(1)
C(3)	3686(2)	7244(2)	5897(1)	18(1)
C(4)	3095(1)	6437(2)	5597(1)	18(1)
Cl(1)	1783(1)	6595(1)	5604(1)	28(1)
C(5)	3459(1)	5278(2)	5251(1)	19(1)
Cl(2)	3264(1)	5692(1)	4644(1)	30(1)
C(6)	3842(1)	3927(2)	5391(1)	18(1)
Cl(3)	4161(1)	2523(1)	4971(1)	30(1)
C(7)	3914(1)	3461(2)	5904(1)	17(1)
Cl(4)	2925(1)	2268(1)	6082(1)	28(1)
C(8)	4623(2)	3923(2)	6215(1)	18(1)
C(9)	4596(1)	3738(2)	6736(1)	17(1)
C(10)	5296(1)	4342(2)	7039(1)	16(1)
C(11)	5091(1)	4522(2)	7565(1)	15(1)
C(12)	4637(1)	3384(2)	7844(1)	19(1)
C(13)	4508(2)	3605(3)	8339(1)	23(1)
C(14)	4827(2)	4953(3)	8556(1)	26(1)
C(15)	5263(2)	6101(2)	8280(1)	23(1)
C(16)	5396(1)	5914(2)	7782(1)	17(1)
C(17)	5877(1)	7035(2)	7443(1)	16(1)
C(18)	6659(1)	6241(2)	7145(1)	17(1)
C(19)	7662(2)	6677(2)	7091(1)	23(1)
C(20)	8282(2)	5890(3)	6770(1)	27(1)
C(21)	7914(2)	4651(3)	6508(1)	25(1)
C(22)	6935(2)	4149(2)	6579(1)	20(1)
C(23)	6299(1)	4928(2)	6900(1)	15(1)
C(24)	5471(1)	8390(2)	7301(1)	16(1)
C(25)	4485(2)	9019(2)	7472(1)	17(1)
C(26)	4235(2)	9320(2)	7955(1)	20(1)
C(27)	3300(2)	9933(2)	8074(1)	23(1)
C(28)	2605(2)	10278(2)	7714(1)	24(1)
C(29)	2827(2)	9959(2)	7233(1)	21(1)
C(30)	3756(1)	9321(2)	7109(1)	17(1)
C(31)	5075(1)	9265(2)	6477(1)	17(1)
C(32)	5337(2)	9832(2)	6020(1)	20(1)
C(33)	6320(2)	10271(2)	5923(1)	25(1)
C(34)	7042(2)	10204(2)	6288(1)	25(1)
C(35)	6789(2)	9675(2)	6749(1)	21(1)
C(36)	5815(1)	9154(2)	6844(1)	17(1)
C(37)	8787(2)	7659(3)	4959(1)	46(1)
Cl(5)	8574(1)	9636(1)	4879(1)	58(1)
Cl(6)	9822(1)	7046(1)	4612(1)	61(1)
Cl(7)	8965(1)	7214(1)	5569(1)	81(1)

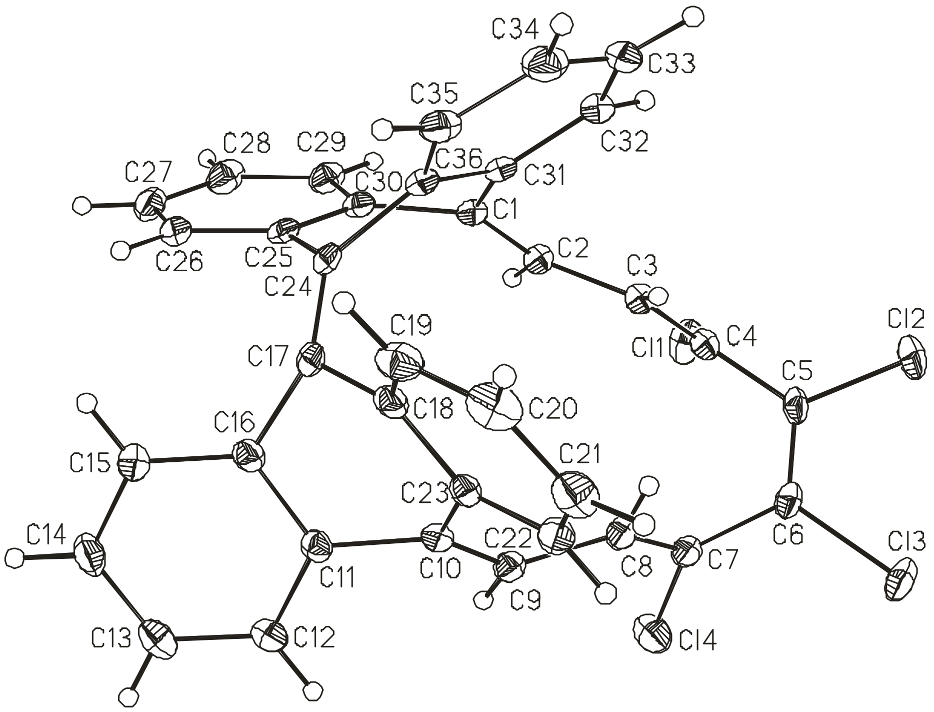

Table S32: Bond lengths [Å].

C(1)-C(2)	1.350(3)	C(17)-C(24)	1.355(3)
C(1)-C(31)	1.475(3)	C(17)-C(18)	1.496(3)
C(1)-C(30)	1.486(3)	C(18)-C(19)	1.391(3)
C(2)-C(3)	1.452(3)	C(18)-C(23)	1.411(3)
C(3)-C(4)	1.339(3)	C(19)-C(20)	1.392(3)
C(4)-C(5)	1.472(3)	C(20)-C(21)	1.386(3)
C(4)-Cl(1)	1.747(2)	C(21)-C(22)	1.385(3)
C(5)-C(6)	1.339(3)	C(22)-C(23)	1.400(3)
C(5)-Cl(2)	1.7332(19)	C(24)-C(25)	1.495(3)
C(6)-C(7)	1.478(3)	C(24)-C(36)	1.499(3)
C(6)-Cl(3)	1.7374(19)	C(25)-C(26)	1.400(3)
C(7)-C(8)	1.335(3)	C(25)-C(30)	1.419(3)
C(7)-Cl(4)	1.744(2)	C(26)-C(27)	1.390(3)
C(8)-C(9)	1.448(3)	C(27)-C(28)	1.388(3)
C(9)-C(10)	1.357(3)	C(28)-C(29)	1.390(3)
C(10)-C(23)	1.477(3)	C(29)-C(30)	1.395(3)
C(10)-C(11)	1.486(3)	C(31)-C(32)	1.399(3)
C(11)-C(12)	1.393(3)	C(31)-C(36)	1.414(3)
C(11)-C(16)	1.412(3)	C(32)-C(33)	1.385(3)
C(12)-C(13)	1.392(3)	C(33)-C(34)	1.391(3)
C(13)-C(14)	1.385(3)	C(34)-C(35)	1.396(3)
C(14)-C(15)	1.385(3)	C(35)-C(36)	1.395(3)
C(15)-C(16)	1.397(3)		
C(16)-C(17)	1.494(3)		
C(37)-Cl(7)	1.745(3)	C(37)-Cl(5)	1.759(3)
C(37)-Cl(6)	1.757(3)		

Table S33: Bond angles [°].

C(2)-C(1)-C(31)	125.14(17)	C(16)-C(17)-C(18)	109.88(16)
C(2)-C(1)-C(30)	121.32(17)	C(19)-C(18)-C(23)	119.55(18)
C(31)-C(1)-C(30)	113.37(16)	C(19)-C(18)-C(17)	126.67(18)
C(1)-C(2)-C(3)	123.14(18)	C(23)-C(18)-C(17)	113.78(16)
C(4)-C(3)-C(2)	127.14(18)	C(18)-C(19)-C(20)	120.0(2)
C(3)-C(4)-C(5)	124.75(18)	C(21)-C(20)-C(19)	120.49(19)
C(3)-C(4)-Cl(1)	122.34(15)	C(22)-C(21)-C(20)	120.10(19)
C(5)-C(4)-Cl(1)	112.84(14)	C(21)-C(22)-C(23)	120.17(19)
C(6)-C(5)-C(4)	122.56(17)	C(22)-C(23)-C(18)	119.47(17)
C(6)-C(5)-Cl(2)	121.28(15)	C(22)-C(23)-C(10)	122.73(18)
C(4)-C(5)-Cl(2)	115.94(15)	C(18)-C(23)-C(10)	117.45(16)
C(5)-C(6)-C(7)	122.92(17)	C(17)-C(24)-C(25)	125.08(17)
C(5)-C(6)-Cl(3)	121.17(15)	C(17)-C(24)-C(36)	120.65(17)
C(7)-C(6)-Cl(3)	115.55(14)	C(25)-C(24)-C(36)	111.78(16)
C(8)-C(7)-C(6)	125.41(18)	C(26)-C(25)-C(30)	118.50(18)
C(8)-C(7)-Cl(4)	121.86(16)	C(26)-C(25)-C(24)	125.35(18)
C(6)-C(7)-Cl(4)	112.68(14)	C(30)-C(25)-C(24)	116.15(16)
C(7)-C(8)-C(9)	126.02(19)	C(27)-C(26)-C(25)	120.60(19)
C(10)-C(9)-C(8)	123.61(18)	C(28)-C(27)-C(26)	120.46(18)
C(9)-C(10)-C(23)	126.19(17)	C(27)-C(28)-C(29)	120.04(19)
C(9)-C(10)-C(11)	121.27(17)	C(28)-C(29)-C(30)	120.1(2)
C(23)-C(10)-C(11)	112.51(16)	C(29)-C(30)-C(25)	120.21(18)
C(12)-C(11)-C(16)	119.99(17)	C(29)-C(30)-C(1)	123.08(18)
C(12)-C(11)-C(10)	123.05(18)	C(25)-C(30)-C(1)	116.69(17)
C(16)-C(11)-C(10)	116.95(16)	C(32)-C(31)-C(36)	119.90(18)
C(13)-C(12)-C(11)	119.95(19)	C(32)-C(31)-C(1)	122.18(18)
C(14)-C(13)-C(12)	120.35(19)	C(36)-C(31)-C(1)	117.75(17)
C(15)-C(14)-C(13)	120.03(19)	C(33)-C(32)-C(31)	120.35(19)
C(14)-C(15)-C(16)	120.84(19)	C(32)-C(33)-C(34)	119.85(19)
C(15)-C(16)-C(11)	118.81(18)	C(33)-C(34)-C(35)	120.53(19)
C(15)-C(16)-C(17)	126.46(18)	C(36)-C(35)-C(34)	120.2(2)
C(11)-C(16)-C(17)	114.68(16)	C(35)-C(36)-C(31)	119.06(18)
C(24)-C(17)-C(16)	125.46(17)	C(35)-C(36)-C(24)	125.82(18)
C(24)-C(17)-C(18)	121.20(17)	C(31)-C(36)-C(24)	115.03(16)
Cl(7)-C(37)-Cl(6)	110.72(17)	Cl(6)-C(37)-Cl(5)	110.79(15)
Cl(7)-C(37)-Cl(5)	111.13(17)		

Table S34: Anisotropic displacement parameters ($Å^2 \times 10^3$). The anisotropic displacement factor exponent takes the form: $-2\pi^2[h^2 a^{*2}U_{11} + ... + 2 h k a^* b^* U_{12}]$.

	U_{11}	U_{22}	U_{33}	U_{23}	U_{13}	U_{12}
C(1)	24(1)	11(1)	17(1)	0(1)	-1(1)	1(1)
C(2)	22(1)	15(1)	16(1)	-1(1)	-1(1)	1(1)
C(3)	23(1)	16(1)	15(1)	0(1)	0(1)	1(1)
C(4)	21(1)	18(1)	15(1)	2(1)	-1(1)	1(1)
Cl(1)	22(1)	38(1)	25(1)	-7(1)	-5(1)	4(1)
C(5)	21(1)	25(1)	10(1)	-2(1)	-2(1)	-5(1)
Cl(2)	43(1)	37(1)	11(1)	2(1)	-3(1)	2(1)
C(6)	19(1)	21(1)	14(1)	-6(1)	-1(1)	-2(1)
Cl(3)	40(1)	28(1)	21(1)	-12(1)	2(1)	4(1)
C(7)	21(1)	16(1)	16(1)	-1(1)	-2(1)	2(1)
Cl(4)	31(1)	29(1)	23(1)	4(1)	-7(1)	-12(1)
C(8)	21(1)	16(1)	16(1)	-3(1)	-1(1)	1(1)
C(9)	19(1)	15(1)	17(1)	-1(1)	-2(1)	-1(1)
C(10)	19(1)	13(1)	15(1)	2(1)	-1(1)	2(1)
C(11)	14(1)	17(1)	15(1)	2(1)	-3(1)	1(1)
C(12)	19(1)	18(1)	20(1)	4(1)	-2(1)	-1(1)
C(13)	22(1)	27(1)	20(1)	8(1)	2(1)	0(1)
C(14)	33(1)	31(1)	14(1)	3(1)	1(1)	4(1)
C(15)	31(1)	21(1)	16(1)	-2(1)	-3(1)	0(1)
C(16)	18(1)	17(1)	15(1)	2(1)	-3(1)	1(1)
C(17)	20(1)	17(1)	12(1)	-3(1)	-5(1)	-5(1)
C(18)	19(1)	16(1)	15(1)	4(1)	-4(1)	1(1)
C(19)	20(1)	20(1)	27(1)	5(1)	-5(1)	-3(1)
C(20)	17(1)	29(1)	35(1)	8(1)	-1(1)	2(1)
C(21)	21(1)	29(1)	26(1)	6(1)	2(1)	9(1)
C(22)	23(1)	19(1)	18(1)	0(1)	-2(1)	5(1)
C(23)	17(1)	16(1)	14(1)	2(1)	-4(1)	2(1)
C(24)	21(1)	14(1)	14(1)	-4(1)	-2(1)	-4(1)
C(25)	24(1)	10(1)	18(1)	-3(1)	1(1)	-5(1)
C(26)	29(1)	14(1)	17(1)	-1(1)	0(1)	-5(1)
C(27)	32(1)	20(1)	18(1)	-6(1)	8(1)	-8(1)
C(28)	23(1)	21(1)	28(1)	-6(1)	8(1)	-4(1)
C(29)	22(1)	17(1)	24(1)	-3(1)	1(1)	-3(1)
C(30)	22(1)	11(1)	18(1)	-1(1)	2(1)	-3(1)
C(31)	24(1)	8(1)	18(1)	-2(1)	1(1)	0(1)
C(32)	29(1)	14(1)	18(1)	0(1)	1(1)	0(1)
C(33)	38(1)	15(1)	20(1)	1(1)	9(1)	-5(1)
C(34)	26(1)	19(1)	31(1)	-1(1)	7(1)	-6(1)
C(35)	24(1)	14(1)	26(1)	0(1)	1(1)	-3(1)
C(36)	23(1)	10(1)	18(1)	-2(1)	1(1)	0(1)
C(37)	48(2)	48(2)	43(2)	7(1)	-15(1)	-20(1)
Cl(5)	60(1)	46(1)	67(1)	2(1)	-4(1)	-6(1)
Cl(6)	72(1)	52(1)	61(1)	2(1)	-4(1)	-1(1)
Cl(7)	103(1)	97(1)	43(1)	22(1)	-13(1)	-18(1)

Table S35: Hydrogen coordinates ($\times 10^4$) and isotropic displacement parameters ($Å^2 \times 10^3$).

	x	y	z	U(eq)
H(2)	2656(17)	7980(30)	6434(8)	20(5)
H(3)	4420(17)	7180(20)	5852(8)	14(5)
H(8)	5153(19)	4530(30)	6084(9)	27(6)
H(9)	3983(18)	3290(30)	6866(9)	24(6)
H(12)	4421	2477	7699	23
H(13)	4205	2843	8524	28
H(14)	4748	5088	8888	31
H(15)	5470	7007	8427	27
H(19)	7917	7494	7270	27
H(20)	8948	6197	6730	33
H(21)	8326	4158	6285	30
H(22)	6700	3291	6414	24
H(26)	4698	9108	8199	24
H(27)	3138	10114	8396	28
H(28)	1990	10722	7795	29
H(29)	2355	10172	6993	25
H(32)	4849	9914	5780	24
H(33)	6496	10610	5616	29
H(34)	7700	10513	6224	30
H(35)	7271	9669	6994	26
H(37)	8189	7107	4845	55
